# Follicular fluid extracellular vesicles improve bovine oocyte quality via lipid and mitochondrial modulation

**DOI:** 10.3389/fvets.2025.1703475

**Published:** 2026-01-14

**Authors:** Fernanda Schneberger, Alessandra Bridi, Priscila Assis Ferraz, José Roberto Quirino de Oliveira, Lorena Calixto Munhoz, Juliano Rodrigues Sangalli, Cibele Maria Prado, Ludimila Cardoso Zoccal Janini, Felipe Perecin, Juliano Coelho da Silveira, Cláudia Lima Verde Leal

**Affiliations:** Departamento de Medicina Veterinária, Faculdade de Zootecnia e Engenharia de Alimentos, Universidade de São Paulo, Pirassununga, Brazil

**Keywords:** bovine, extracellular vesicles, follicular fluid, *in vitro* maturation, lipid droplet, mitochondria, oocyte

## Abstract

*In vitro* maturation (IVM) systems remain sub-optimal, compromising oocyte cytoplasmic competence and subsequent embryo quality by failing to reproduce follicular fluid (FF) microenvironment communication, specifically by lacking extracellular vesicles (ffEVs). In order to determine the contribution of ffEVs on oocyte maturation and quality, oocytes were assessed for nuclear maturation, mitochondrial activity, reactive oxygen species (ROS) levels, and lipid metabolism parameters such as lipid droplet (LD) area, HSL phosphorylation and glycerol release levels, after IVM of COC with or without ffEVs. Subsequent embryo development rates and blastocyst quality (total cell number, mitochondrial activity and LD area) were also evaluated. Additionally, the relative abundance of mRNA and miRNAs that regulate lipid metabolism as well as transcripts of antioxidant enzymes were also analyzed in oocytes, cumulus cells (CC), and ffEVs. While nuclear maturation was unaffected, ffEVs supplementation significantly improved cytoplasmic competence, evidenced by increased mitochondrial activity and lower ROS levels. Despite lipolytic activity being unchanged, the oocyte LD area increased implying enhanced lipogenesis and/or lipid uptake independent of lipolytic activity. These effects did not alter early embryonic development rates and total cell number in Day 7 blastocysts. Nonetheless, blastocyst mitochondrial activity and LD area reduced, indicating that energy and lipid metabolism are being modulated in a beneficial way, favoring embryonic quality. Although many candidates lipid-regulating miRNAs and mRNAs, as well as antioxidant enzyme mRNAs, were detected in ffEVs, their expression in oocytes and CC was unchanged after IVM. Only bta-miR-23b-3p was downregulated in CC. In conclusion, ffEVs induce favorable metabolic alterations in the oocyte, increasing oocyte quality and improving blastocyst lipid contents. This shows ffEVs as a promising additive tool to enhance IVM condition in bovine embryo IVP systems.

## 1 Introduction

In vitro production of bovine embryos (IVP) is important for advancing genetic improvement and increasing animal fertility. This technology depends on the attainment of oocyte competence, described as the intrinsic ability of the oocyte to undergo nuclear and cytoplasmic maturation, successfully fertilize, and develop to the blastocyst stage (1). *In vivo*, oocyte development and maturation occur through complex bidirectional communication between the oocyte and neighboring cumulus cells (CC) and granulosa cells (2), which includes the exchange of extracellular vesicles (EVs) within the follicular fluid (FF) that surrounds the cumulus-oocyte complexes (COCs). The interactions between these cells play a regulatory role in oocyte quality (3). Despite advancements, IVP efficiency remains inferior to *in vivo* conditions, reflecting in lower blastocyst formation and pregnancy rates, partly due to compromised oocyte quality resultant from suboptimal culture environment (4–6). This disparity drives the effort for a better understanding of the mechanisms which influence oocyte competence in order to optimize culture conditions.

In the ovarian follicle, intercellular interactions occur mediated by the direct transfer of molecules through the transzonal projections (TZP) connecting CC and the oocyte (7), and by factors supplied by the FF (8, 9). The composition of the FF varies with follicular stage and estrous cycle (10), influencing oocyte quality and developmental competence (11). Consequently, the disruption of this intrafollicular communication, as occurs during IVM when oocytes are removed from the follicular environment and placed in suboptimal culture media, negatively impacts oocyte competence (12–14). This bottleneck has shifted focus toward utilizing components of the FF to provide a more physiological environment *in vitro* (15, 16). Among the components of FF, EVs (ffEVs) are membranous nanostructures released by cells of the ovarian follicle carrying a complex cargo of RNA, proteins, and lipids, serving as essential mediators of intercellular communication (15, 17, 18). In the context of bovine reproduction, ffEVs have been shown to be internalized by CC and subsequently trafficked to the oocytes via TZP (16, 19). Supplementation of IVM media with ffEVs has been shown to support cumulus expansion, increase nuclear maturation, fertilization, and improve blastocyst yield and quality (18, 20–23). The presence of specific miRNAs in FF and within ffEVs was associated with better oocyte quality and greater embryonic development potential (24–27). Interestingly, removing EVs naturally present in serum and replacing them with EVs from FF, oviduct, and uterus confirms the modulatory role of EVs during *in vitro* culture (16, 19, 28, 29).

Besides the general modulation of oocyte development, ffEVs may act directly on key determinants of oocyte quality as energy metabolism and organelle function (30, 31). Bovine oocytes contain cytoplasmic lipid droplets (LDs), which serve as an energy reserve utilized via *β*-oxidation during maturation and early development (32, 33). Impaired *β*-oxidation or dysregulated lipid metabolism leads to excessive fatty acid accumulation, inducing lipotoxicity, oxidative stress and apoptosis, thereby compromising both oocyte and embryonic competence, as evidenced by lower blastocyst rates and reduced cryotolerance (34–36). Furthermore, mitochondria, central to ATP production and cellular redox balance, impact oocyte quality (37). Accordingly, mitochondrial dysfunction and elevated reactive oxygen species (ROS) levels impair both oocyte maturation and subsequent embryonic development *in vitro* (38–40). EVs have been shown to modulate mitochondrial function and mitigate oxidative stress by transferring mitochondrial components (41–43). Modulation of lipid contents in embryos by oviductal and uterine EVs has also been reported (29). The proteome of bovine ffEVs was shown to contain proteins related to mitochondrial function, antioxidant defense and lipid metabolism (18). This evidence highlights the potential role of ffEVS in integrating lipid metabolism, mitochondrial activity and antioxidant defense, mechanisms vital for oocyte competence.

To investigate the hypothesis that ffEVs enhances oocyte quality, the present study investigated the impact of ffEVs supplementation during IVM on oocyte cytoplasmic competence, focusing on the modulation of lipid metabolism, mitochondrial activity, and ROS levels, and evaluated the carry-over effects of these metabolic shifts on the resultant blastocyst quality. Additionally, we aimed to correlate the phenotypic effects with the presence in ffEVs cargo of selected mRNAs and miRNAs involved in lipid metabolism and antioxidant defense and modulation of their relative abundance in the oocytes and CC matured with ffEVs.

## 2 Materials and methods

### 2.1 Ethics

No experiments involving living animals were performed. All procedures were approved by the Animal Care and Use Committee (CEUA, Protocol 1923030223) of the Faculty of Animal Science and Food Engineering of the University of São Paulo (FZEA/USP).

### 2.2 Chemicals

All chemicals were purchased from Sigma-Aldrich (Saint Quentin Fallavier, France), unless otherwise specified.

### 2.3 Experimental design

**Figure 1:**
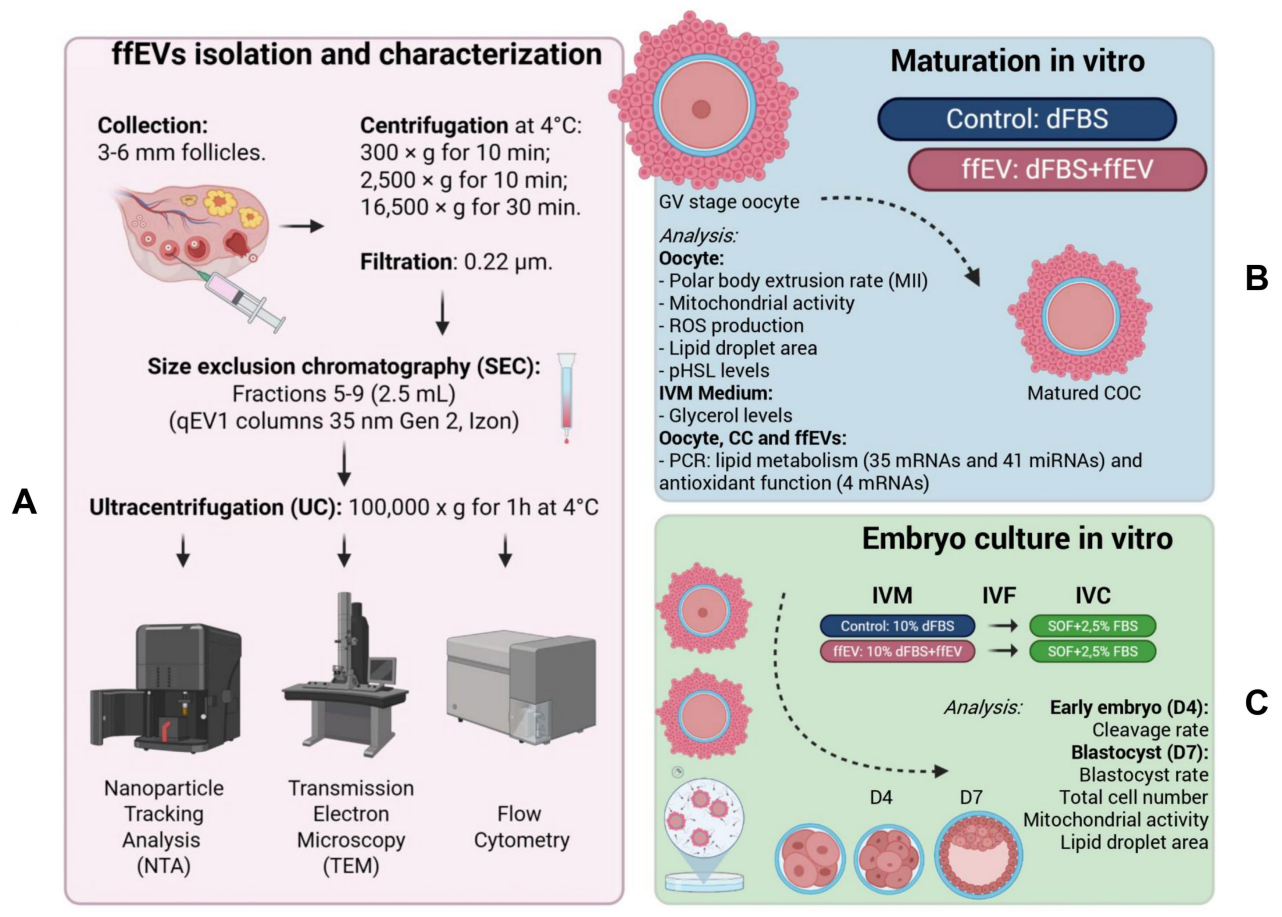
Experimental design. **(A)** Follicular fluid extracellular vesicles (ffEVs) were isolated from ovarian follicles measuring 3 to 6 mm in diameter through a series of differential centrifugation steps performed at 4 °C, including spins at 300 × *g* for 10 min, 2,500 × *g* for 10 min, and 16,500 × *g* for 30 min, followed by filtration through a 0.22 μm membrane to remove cellular debris. The vesicle-containing fractions (5–9, approximately 2.5 mL) were subsequently purified using size exclusion chromatography (SEC) with qEV1 columns (35 nm Gen 2, Izon), and concentrated by ultracentrifugation at 100,000 × *g* for 1 h at 4 °C. The isolated ffEVs were characterized for size, concentration, and morphology using nanoparticle tracking analysis (NTA), transmission electron microscopy (TEM), and flow cytometry. **(B)** To evaluate the functional impact of ffEVs on oocyte maturation, IVM was conducted under two conditions: Control, in vitro maturation medium supplemented with 10% EVs-depleted fetal bovine serum (dFBS) only, and ffEV, the same medium additionally supplemented with ffEVs (3.0 × 10^10^ particles/mL, equivalent to 10% concentration of FF). Following IVM, parameters were assessed in oocyte, including first polar body extrusion (PBE) rate, mitochondrial activity, ROS production, lipid droplet area, phosphorylated hormone-sensitive lipase (pHSL) levels. Oocytes, CC and ffEVs were evaluated for gene expression profiles associated with lipid metabolism (35 mRNAs and 41 miRNAs) and antioxidant functions (4 mRNAs) via RT-qPCR analysis. **(C)** IVF and embryo culture were performed with oocytes matured in either control or ffEV-supplemented conditions; embryos were cultured in synthetic oviductal fluid (SOF) with 2.5% FBS. Embryonic development was evaluated by cleavage rate at day 4 and blastocyst formation at day 7, along with total cell numbers, mitochondrial activity, and lipid droplet area in blastocysts.

The study was structured in two main experiments (Figure 1). A preliminary phase was carried out to isolate and characterize ffEVs. Experiment 1 assessed how ffEVs supplementation during IVM affected oocyte maturation, mitochondrial activity, ROS levels and lipid metabolism, as well as subsequent embryo development and quality. In Experiment 2, RNA expression was examined by comparing the mRNA and miRNA profiles associated with lipid metabolism in oocytes and CC matured with or without ffEVs, and by relating these profiles to the RNA cargo contained within ffEVs.

### 2.4 Collection of follicular fluid

Bovine ovaries were collected from a commercial slaughterhouse and transported in 0.9% NaCl saline at ambient temperature (37 °C). The time between collection and sample processing was a maximum of 3 h. FF was aspirated from 3–6 mm follicles using an 18-gage needle and 10 mL syringe, then transferred to 1.5 mL microtubes. Only ovaries without visible abnormalities, lacking follicles larger than 10 mm, and containing only recently ovulated corpora lutea were selected, following criteria established by Ireland et al. (44). FF samples, ranging from 1 to 16 mL, were pooled from 2 to 20 ovaries. To purify the fluid, FF samples underwent three sequential differential centrifugations at 4 °C: 300 × *g* for 10 min (to remove cells), 2,500 × *g* for 10 min (to remove cellular debris), and 16,500 × *g* for 30 min (to remove apoptotic bodies and microvesicles). The resultant supernatant was filtered (0.22 μm) and stored at −80 °C. In total, 40 FF samples were collected from 140 ovaries.

### 2.5 Measurements of estradiol and progesterone in follicular fluid samples

Estradiol (E2) and Progesterone (P4) concentrations were measured via chemiluminescence (ADVIA Centaur-Siemens) at Pasin Laboratory in Santa Maria, Brazil. A 200 μL FF aliquot was diluted 1:50 in PBS to ensure detection within the hormonal assay’s limit. Follicles were categorized as healthy (>1), transitional (1–0.01), or atretic (<0.01) based on their E2:P4 ratio. For *in vitro* culture experiments, only ten FF samples from transitional follicles (E2:P4 ratio 0.1–1) were selected. Intra-assay coefficients of variation were 19.78% for P4 and 13.77% for E2.

### 2.6 Isolation of extracellular vesicles from follicular fluid

The ffEV samples were thawed on ice and isolated via size exclusion chromatography (SEC) using Izon qEV1 35 nm Gen 2 columns, following manufacturer instructions. Columns were washed with ≥ 20 mL PBS at room temperature (22 °C) to remove storage solution. After drainage, FF samples were loaded. Upon sample entry, 10 mL of PBS was added for elution, initiating 0.7 mL fraction collection. The first fractions constituted the void volume (~4.7 mL). Subsequently, purified EV-rich fractions, totaling 2.5 mL, were collected. EV-rich fractions were concentrated by ultracentrifugation at 100,000 × *g* for 70 min at 4 °C (Optima XE-90 Ultracentrifuge, 70 Ti rotor, Beckman Coulter, United States). Resulting pellets were resuspended in either calcium- and magnesium-free 1 × PBS for characterization or TCM199 for *in vitro* culture experiments, and stored at −80 °C.

### 2.7 Nanoparticle tracking analysis of extracellular vesicles

Nanoparticle Tracking Analysis (NTA) was conducted to determine vesicle size and concentration across samples. A 100 μL ffEVs sample, isolated from 1 mL of FF, was utilized for NTA. All measurements were performed using the NanoSight NS300 system (Malvern Technologies, Malvern, UK) equipped with a high-sensitivity sCMOS camera. To quantify, samples were diluted twice in calcium- and magnesium-free 1 × PBS at 5:995 and 100:900 or 10:990 and 50:950 ratios. Data processing was performed with NTA 3.4 Build 3.4.003 software, capturing five 30-s videos per sample. Analysis parameters included a Detection Threshold of 3–5, a Camera Level of 13–14, a temperature of 38.5 °C, and a dynamic viscosity of 0.67 cP.

### 2.8 Transmission electron microscopic analysis of extracellular vesicles

For Transmission Electron Microscopic (TEM) analysis, ffEVs were isolated from 1 mL of FF. Pelleted EVs were resuspended in fixative solution (0.1 M cacodylate, 2% glutaraldehyde, 2% paraformaldehyde; pH 7.2–7.4) and incubated 2 h at room temperature. Fixative removal involved 2 mL PBS addition, then ultracentrifugation (100,000 × *g*; 70 min; 4 °C). The pellet, resuspended in 200 μL Milli-Q water, was refrigerated until analysis at the Multiuser Electron Microscopy Laboratory of the Department of Cellular and Molecular Biology, Faculty of Medicine of Ribeirão Preto, USP, Brazil. Samples were placed on a pioloform-coated copper grid, air-dried for 30 min, and stained with 2% aqueous uranyl acetate for 3 min. Excess stains were removed with filter paper. Imaging utilized JEM 100X II (JEOL, Japan).

### 2.9 Flow cytometry analysis of extracellular vesicles

To characterize specific EV proteins and confirm isolation purity, flow cytometry (CytoFLEX, Beckman Coulter, United States), utilizing 405 nm and 488 nm lasers, was employed. 1 mL of FF were used to isolate ffEVs (1,011 particles/mL), that were diluted in 400 μL PBS. For each marker, a 100 uL aliquot of ffEVs was taken. Sample was subjected to incubation with primary antibodies targeting tetraspanins, including CD81 (FITC-conjugated mouse monoclonal, AB239256, 1:20) and CD9 (FITC-conjugated mouse monoclonal, AB18241; 1:50). Syntenin (primary antibody SC515538, 1:100; followed by Alexa Fluor 488 goat anti-mouse polyclonal secondary antibody A11001, 1:200) was also detected. For permeabilization prior to Syntenin and Calnexin antibody incubation, samples were treated with 0.001% Triton X-100™ (1:1 dilution) for 15 min at room temperature. Calcein-AM (Sigma-Aldrich; 17,783; 1:100) served as a marker for cytoplasm-containing nanoparticles. Calnexin (AF488; 1:50), an endoplasmic reticulum protein, was used as a negative control to detect cellular contamination. Incubation periods varied: Calcein and Calnexin were incubated for 20 min at 37 °C, CD81 and CD9 for 2 h at room temperature, and Syntenin primary antibody for 30 min followed by secondary antibody for 1 h 30 min at room temperature. Negative controls, consisting of PBS combined with the respective antibodies but without ff EVs, exhibited a low signal, confirming staining specificity and minimal non-specific binding.

### 2.10 Collection of cumulus-oocyte complexes and *in vitro* maturation

Bovine ovaries from 10 biological replicates were collected from commercial slaughterhouses and transported to the laboratory in 0.9% NaCl physiological saline solution at ambient temperature. Upon arrival, ovaries were washed in saline and kept in a thermos flask. Follicles 3–8 mm in diameter were aspirated using a syringe and needle. The follicular fluid was collected, allowed to decant, and the supernatant was centrifuged. COCs were retrieved from the decanted pellet and placed in Petri dishes for selection. Only Grade I or II COCs, characterized by homogeneous cytoplasm and at least 3–4 compact CC layers, were selected. COCs were matured in groups of 40–50 in 500 μL maturation medium for 21–23 h at 38.5 °C in a humidified atmosphere with 5% CO₂ in air. The medium consisted of TCM199 with Earle’s salts and 0.4 mM L-glutamine, supplemented with 10% EV-depleted FBS, 22 μg/mL pyruvate, 0.5 μg/mL FSH (Folltropin®, Vetoquinol, France), 50 μg/mL gentamicin, and 10 ng/mL EGF. For EV-supplemented conditions, ffEVs were added to the IVM medium at 3.0 × 10^10^ particles per mL (10% proportion), based on the method by Silveira et al. (24). EVs-depleted FBS (dFBS) was obtained by ultracentrifugation (100,000 × *g* for 18 h at 4oC) of a pool of five batches of FBS (GIBCO), and collection of the supernatant which was aliquoted and stored at -20oC to be used for all experiments.

### 2.11 *In vitro* fertilization and embryo culture

*In vitro* fertilization (IVF) of COCs was conducted following IVM, in 10 biological replicates. COCs were initially washed in IVF medium (Fert-TALP) and transferred to 90 μL droplets of the same medium, overlaid with sterile mineral oil. Semen from a single bull (OPIO CFM, batch 201011-2) was prepared by thawing at 36 °C for 30 s and subjected to a 45% over 90% Percoll gradient, centrifuged at 4,500 rpm for 7 min. The supernatant was discarded, and the viable sperm pellet was resuspended in 500 μL IVF medium. A subsequent washing step involved centrifugation at 2,500 rpm for 5 min, after which the pellet was resuspended in 30 μL IVF medium. Sperm motility and concentration were assessed to achieve a final concentration of approximately 1 × 106 spermatozoa/mL. Six μL of this sperm suspension was then added to each IVF droplet containing the pre-washed COCs. Gamete co-incubation occurred for 6 h at 38.5 °C in a 5% CO₂ in air atmosphere. Following co-incubation, presumptive zygotes were washed three times to remove excess spermatozoa and CC. Embryo culture proceeded in 500 μL SOF with 2.5% FBS and amino acids, within 5-well plates (WTA Technologies, Brazil), maintained at 38.5 °C under a gas atmosphere of 5% CO₂, 5% O₂, and 90% N₂. The cleavage rate was defined as the proportion of oocytes reaching 8 or more cells by day 4, while the blastocyst rate represented the proportion reaching early blastocyst, blastocyst, and expanded blastocyst stages by day 7. Both rates were calculated as the ratio of developed structures to the total number of oocytes cultured.

### 2.12 Determination of maturation rate

Oocyte maturation was assessed by determining nuclear progression to metaphase II. Following IVM, oocytes were denuded and examined via stereomicroscopy for the presence of the first polar body extrusion (PBE). The maturation rate (%PBE) was calculated as the proportion of oocytes exhibiting a visible PBE relative to total evaluated oocytes.

### 2.13 Staining of oocytes and embryos for mitochondrial activity, lipid droplet area, chromatin, and ROS levels

For mitochondrial activity and lipid droplet area rate, samples underwent sequential staining (45, 46). Denuded oocytes (~20 per group, 6 biological replicates) or D7 blastocysts (~12 per group, 4 biological replicates), according to the experiment, were incubated for 30 min at 38.5 °C in 400 nM MitoTracker Orange CMTMRos (Invitrogen, California, United States) and then washed (three times for 5 min) in PBS + 0.1% PVP (PP), followed by fixation in 4% paraformaldehyde for 30 min at room temperature. Next, samples were washed and permeabilized in 0.1% saponin in PP for 30 min. Then, for lipid droplet staining, samples were washed and incubated with 20 μg/mL Bodipy 493/503 (Invitrogen, California, United States) for 1 h. After this step, oocytes were washed and mounted in 10 μL of ProLong Diamond (Thermo Fisher Scientific, Massachusetts, United States). In order to count total cell numbers (TCN) in blastocysts, after the last step, only embryo samples were washed and further stained with 10 μg/mL Hoechst 33342 (Invitrogen, California, United States) for 15 min to visualize chromatin (nuclei). After washing, embryos were mounted as described for oocytes.

For ROS levels, matured COCs were denuded (~20 per group, 4 biological replicates) and incubated for 30 min at 38.5 °C in 5 μM CellROX® Orange (Life Technologies, Saint-Aubin, France). Then, samples were washed three times in PP and mounted in Fluoromount™ and analyzed immediately.

### 2.14 Imaging and quantitative analysis of oocytes and embryos

The images were acquired using MICA microscope (Leica, Wetzlar, Germany), with confocal HyD FS detection set. We used a 63 × objective at a resolution of 2,984 × 2,500 equipped with a diode laser. The laser excitation and emission were set at, respectively, 550 and 573 nm for mitochondria, 493 and 503 nm for lipid droplets and 361 and 497 nm for TCN. All images were captured using the same parameters, performing sequential acquisition. We captured serial ~3 μm confocal images of each sample: one image at the midpoint (the section with the largest diameter) and two additional images from each half, one taken near the middle and the other toward the end of the sample. Physical length (space filled by the sample) was ~12 μm. Based on a previous study that established the correlation between three central sections (47), confirmed in another study (45) that used only the equatorial region of the sample for quantitative measurements, we selected the image at the oocyte or embryo midpoint for analysis (29, 38, 46, 48).

For ROS levels in oocytes, images acquisition was performed using MICA microscope (Leica, Wetzlar, Germany) with a 20 × objective and Incident Fluorescence Illumination (LED) Light Source, in place of the confocal settings. The focus was set on the equatorial region of the samples. All images were captured under the same parameters.

Mitochondrial activity was assessed by measuring fluorescence signal intensity (pixels) in the samples. For lipid quantification, droplet areas were measured and normalized by total oocyte area to account for size variations, while, in blastocysts, besides normalizing by embryo area, to account for size differences, the blastocoel area was subtracted if present, so only the cellular area would be analyzed. Images obtained were segmented using Ilastik (Heidelberg, Germany) software (49, 50), which classifies pixels using machine learning, to separate the area of interest (with fluorescence) and background into distinct objects. The original and segmented images were then input into CellProfiler (Broad Institute, Cambridge, United States) software (49, 51, 52). To measure mitochondrial activity, the “Measure Image Intensity” in “areas enclosed by objects” was set in the pipeline, and the measurement exported was “Total Intensity,” which corresponds to the sum of all pixels intensities within an object, expressed in arbitrary units (a.u.), with software assignment of intensity values between 0 and 255 for each pixel. For lipid droplet area and cytoplasmic area, the “Measure Image Area Occupied” by “Objects” was set in the pipeline, and the measurement exported was “Area Occupied,” which represents the total area occupied by the objects, as the sum of the pixels that constitute these objects, expressed in arbitrary units (a.u.). For TCN analysis in blastocysts, maximum projection was done to enhance visualization of all structures. The cell counting was performed using the “Cell Counter” plugin in ImageJ (NIH, Bethesda, United States).

ROS levels in oocytes were evaluated using ImageJ. After selection of the oocyte area using the freehand selection tool, each oocyte was measured to determine its area and its integrated density (IntDen), which corresponds to pixel intensity. Also, the background fluorescence of an area outside the oocyte was measured. Fluorescence intensity in each oocyte was determined using the following formula: Relative fluorescence = IntDen − (area of selected oocyte x mean fluorescence of background readings). Fluorescence intensities are expressed in arbitrary units (a.u.).

### 2.15 Western blot in oocytes

For western blot analysis, proteins were extracted from pools of 50 oocytes per treatment group across four biological replicates. Protein extraction was performed using RIPA buffer supplemented with protease and phosphatase inhibitors. Lysates were denatured by mixing with 4 × Laemmli sample buffer (Bio-Rad, Hercules, United States) and heating at 95 °C for 5 min. Proteins were separated by 10% SDS-PAGE at 100 V for 90 min and transferred onto PVDF membranes using a Trans-Blot Turbo Transfer System (Bio-Rad, Hercules, United States). Membranes were washed three times with 1 × Tris-buffered saline containing 0.1% Tween-20 (TBST) and blocked with 3% BSA in TBST for 1 h at room temperature. Primary antibodies against active phosphorylated hormone-sensitive lipase (pHSL, Ser563; Cell Signaling Technology, Boston, United States) and *β*-actin as a loading control were incubated overnight at 4 °C with gentle agitation. After three 5-min TBST washes, membranes were incubated for 1 h at room temperature with an HRP-conjugated anti-rabbit secondary antibody (1:5000; A0545; Sigma-Aldrich). Detection was performed following three additional TBST washes using Clarity Western ECL chemiluminescent substrate (170–5,060; Bio-Rad, Hercules, United States). Protein expression was normalized to endogenous *β*-actin, with phosphorylation levels expressed as the pHSL:β-actin ratio. Image of immunoblotting (Supplementary Figure 5) was captured using the ChemiDoc MP Imaging System (Bio-Rad, Hercules, United States).

### 2.16 Measurement of glycerol levels

For glycerol collection, 25 COCs were cultured in 100 uL of IVM medium with or without ffEVs, in 4 biological replicates. Following IVM, samples were collected and frozen at −80 °C. The Glycerol-Glo™ Assay (Promega, Madison, United States) was employed, following manufacturer guidelines, utilizing a standard curve ranging from 0 to 40 μM. For the assay, 30 μL of each sample or standard was combined with 30 μL of Glycerol Detection Reagent in a white 96-well plate. The plate was shaken for 30–60 s, then incubated at room temperature for 1 h. Luminescence was recorded using a FLUOstar OPTIMA (BMG LABTECH, Ortenberg, Germany) for up to 1 h until a signal plateau was achieved. Sample luminescence was converted to glycerol concentration using the luminescence of a known glycerol standard and a 0 μM negative control.

### 2.17 Gene expression analysis

#### 2.17.1 RNA extraction

Samples of oocytes and their CC (replicates of 50 oocytes per group) were stored in 1.5 μL PBS + 0.1% PVP at −80 °C. ffEVs were isolated from 1 mL of FF (3 × 10^11^ particles/mL, pool of 10 biological replicates) and stored in 100 μL PBS at −80 °C. mRNA from oocytes and their CCs (4 biological replicates) was extracted using the PicoPure® RNA Isolation Kit (Thermo Fisher Scientific, Massachusetts, United States). For mRNA and miRNA from ffEVs (pool of 10 samples) and miRNA from oocytes and their CCs (4 biological replicates), total RNA were extracted with TRIzol™ Reagent and GlycoBlue™ Coprecipitant (15 mg/mL). All RNA samples underwent Invitrogen™ DNase I treatment, with quantity and quality assessed by NanoDrop 2000 spectrophotometry (Thermo Fisher Scientific, Massachusetts, United States).

#### 2.17.2 Reverse transcription

For mRNA analysis, cDNA was synthesized using the High Capacity Kit (Thermo Fisher Scientific, Massachusetts, United States). The 20 μL reverse transcription reaction, was incubated 10 min at 25 °C, 120 min at 37 °C, and 5 min at 85 °C. miRNA analysis involved a two-step cDNA conversion: a 5 μL poly(A) tailing reaction with 100 ng total RNA, 10 μM Adapter Primer, and Poly(A) Tailing of RNA using E. coli Poly(A) Polymerase Kit (M0276, New England Biolabs, Ipswich, United States) at 37 °C for 60 min, then 70 °C for 5 min. Followed by a 10 μL reverse transcription using the product and ReadyScript® cDNA Synthesis Mix (25 °C for 5 min, 42 °C for 30 min, then 85 °C for 5 min). Subsequent cDNA samples were immediately used for qRT-PCR. For mRNA analyses of oocytes and CC, cDNA was diluted 8x and 16x, respectively, in RNase/DNase-free water. For ffEV mRNA analysis, 15 ng/mL of mRNA was used per PCR well.

#### 2.17.3 Real-time quantitative polymerase chain reaction

Relative mRNA and miRNA expression were quantified via RT-qPCR. Reactions utilized the A6002 GoTaq® qPCR Master Mix (QIAGEN, Hilden, Germany), with final volumes of 10 μL (mRNA) and 6 μL (miRNA). mRNA reactions combined Master Mix, forward/reverse primers (0.5, 5, or 10 μM), cDNA, and RNase-/DNase-free water. miRNA reactions included Master Mix, 10 μM Universal/Forward Primers, 500 pg. RNA cDNA, and RNase-/DNase-free water. Amplification occurred on a QuantStudio 6 Flex (Thermo Fisher Scientific, Massachusetts, United States). mRNA thermal cycling began with 50 °C for 2 min and 95 °C for 10 min, then 45 cycles of 95 °C for 15 s and 60 °C for 1 min (fluorescence capture). A melting curve (95 °C for 15 s, 60 °C for 1 min, 95 °C for 15 s, final fluorescence capture) confirmed single amplification products. For microRNA PCR, thermal cycling involved an initial activation at 95 °C for 5 min, followed by 45 cycles of 95 °C for 10 s (denaturation), 60 °C for 30 s, and 70 °C for 30 s (annealing/extension), with fluorescence capture occurring during cycling. A melting curve analysis (95 °C for 15 s, 60 °C for 1 min, 95 °C for 15 s, with final fluorescence capture) confirmed single amplification products. mRNA transcripts were considered present if exhibiting a Ct value below 37 and a single melting peak. Their Ct values were normalized using the geometric mean of *ACTB, PPIA, RPL15*, and *RPL8*. For miRNAs, expression was considered if Ct ≤ 37, with up to two melting curve peaks (precursor/mature forms) and presence in a single replicate. miRNA levels in ffEVs were normalized to bta-miR-99b, Hm/Ms./Rt T1 snRNA, and RNT43 snoRNA.

### 2.18 Quantification and statistical analysis

**Figure 2:**
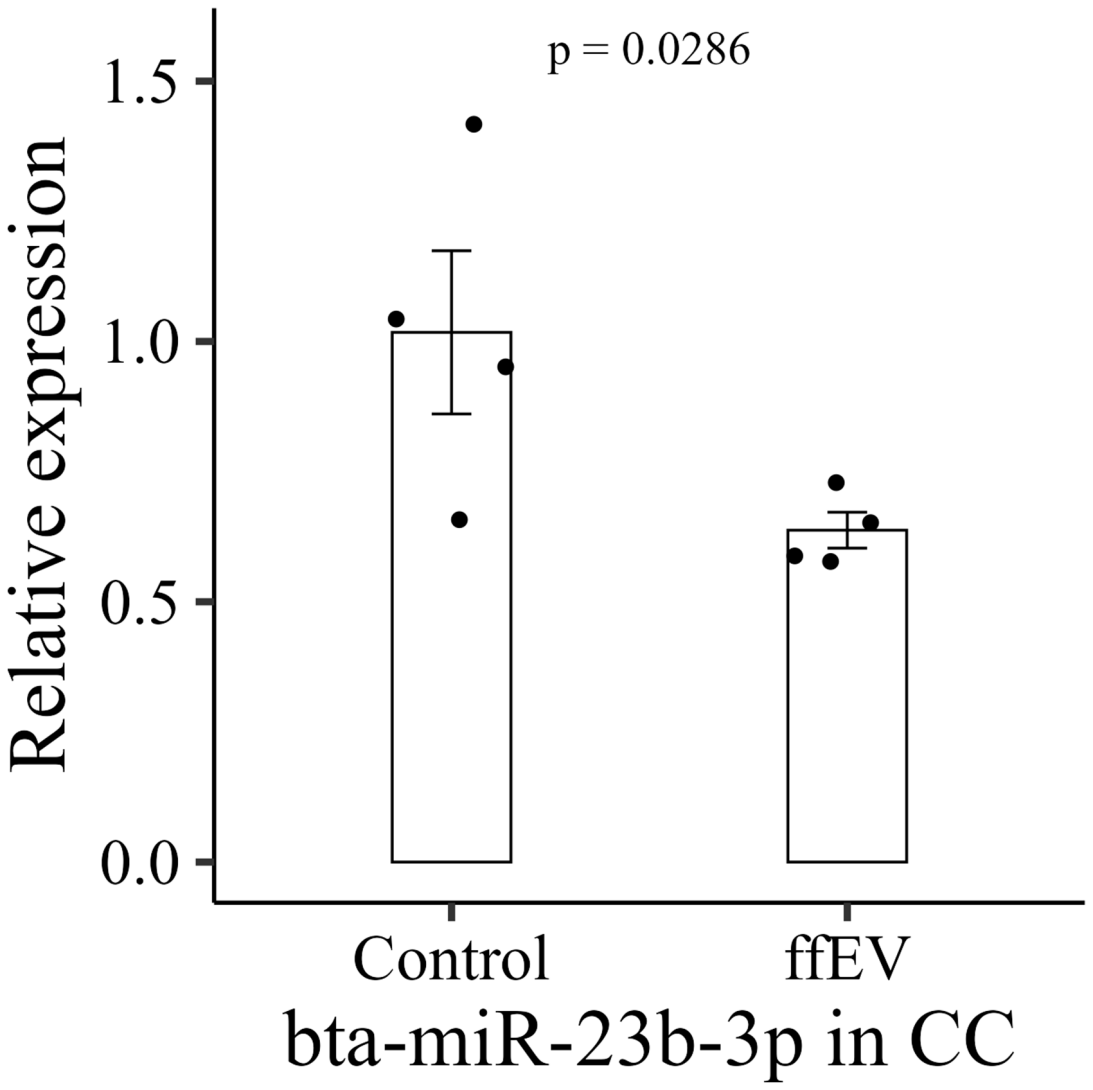
Relative abundance of *bta-miR-23b-3p* in cumulus cells. Data are presented as mean ± standard error of the mean (SEM).

Statistical analysis was performed using the R Studio (Posit, Boston, United States) and JMP (SAS Institute, Cary, United States). Glycerol levels, LD number and proportion data (maturation, cleavage, blastocyst, pHSL:*β*-actin, LD area) were compared by Mann–Whitney Rank Test. Prior to evaluating TCN in embryos and all other fluorescence intensity data, Rosner’s Test for outlier detection was applied. This test identifies multiple outliers by iteratively eliminating the most extreme data points and calculating the Extreme Studentized Deviate (ESD) statistics at each stage. After verifying if the outlier indicated any inconsistencies in the images, the data was removed. Following data cleaning, an analysis of variance (ANOVA) was performed. If the residuals did not exhibit normality (Shapiro–Wilk test) and homoscedasticity (Levene’s test), the Mann–Whitney Rank test was employed. For qPCR, delta-Ct (dCt) data were submitted to Wilcoxon Exact test and plotted as 2^-dCt^ values (Figure 2; Supplementary Figures 2, 3). We employed a 5% (*p* < 0.05) level of significance for all tests.

## 3 Results

### 3.1 Hormonal analyses of follicular fluid

Follicular fluid from 40 samples was analyzed to determine E2 and P4 concentrations, along with E2: P4 ratio (Supplementary Figure 1). From these, 10 samples with an E2: P4 ratio greater than 0.01 were selected for further analysis. The selected samples exhibited mean E2 concentrations of 32.28 ± 5.68 ng/mL and P4 concentrations of 108.75 ± 5.65 ng/mL, yielding an average E2: P4 ratio of 0.31 ± 0.05.

### 3.2 Characterization of small extracellular vesicles obtained from follicular fluid

**Figure 3:**
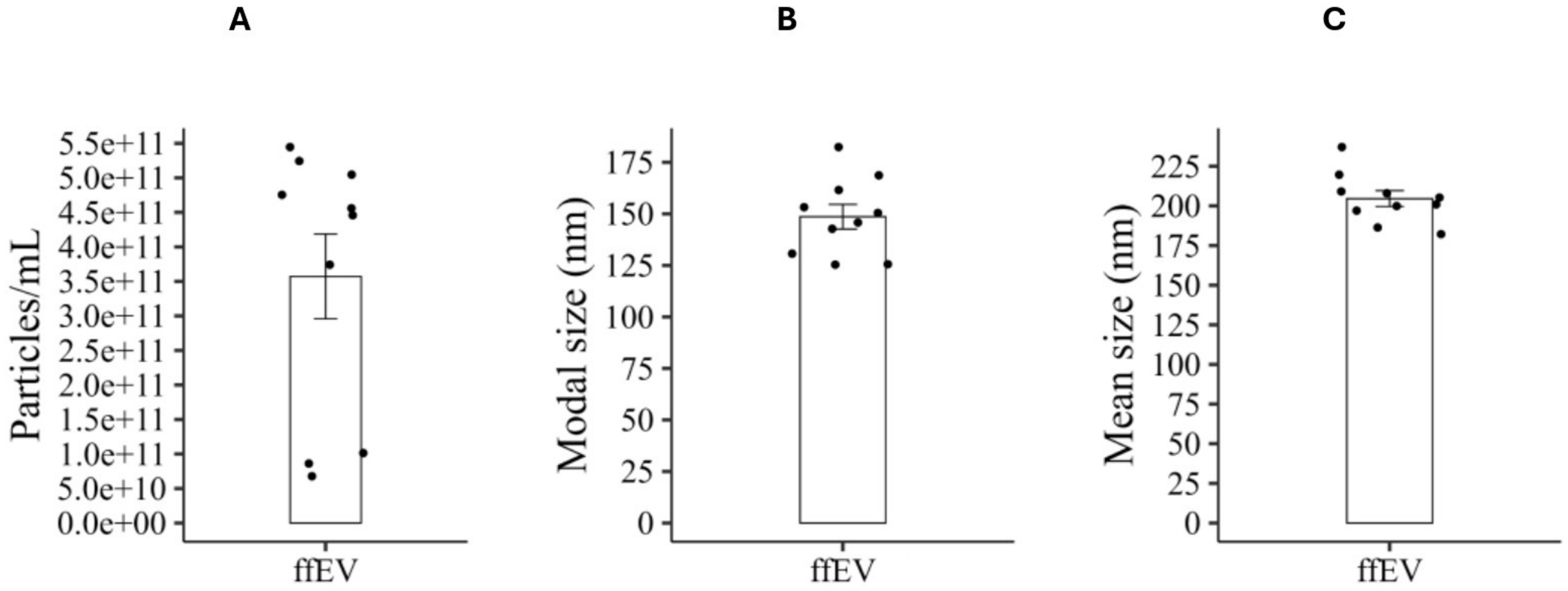
Characterization of ffEV. **(A)** Concentration, **(B)** modal, and **(C)** mean particle size analyzed by NTA from 10 FF samples. Data are presented as mean ± standard error of the mean (SEM).

The ffEVs were characterized using NTA, TEM, and flow cytometry, aligning with Minimal Information for Studies of Extracellular Vesicles (MISEV) guidelines (48). NTA determined a mean particle concentration of 4.5 × 10^11^ ± 1.9 × 10^11^ particles/mL (Figure 3A). The particle size distribution showed a modal diameter of 147.5 ± 18.9 nm (Figure 3B) and a mean diameter of 203.0 ± 15.7 nm (Figure 3C). TEM confirmed the presence of vesicular structures similar to small EVs, characterized by cup-shaped morphology typically associated with exosomes and diameters below 200 nm (Supplementary Figures 4 A,B). Flow cytometry validated EVs identity by detecting characteristic surface and intraluminal markers (Supplementary Figure 4C). Positive EVs markers included CD81 (with Calcein, 218.41 events/μL), CD9 (29.5 events/μL), and Syntenin (13.54 events/μL). Calnexin, a negative marker for cellular contamination, was undetectable (0.0 events/μL), indicating minimal contamination in isolated EVs (19). Negative controls (PBS + antibody without ffEVs) showed low signal, confirming staining specificity and absence of non-specific binding.

### 3.3 Effect of supplementation of small extracellular vesicles during IVM on oocyte maturation and metabolism

**Figure 4:**
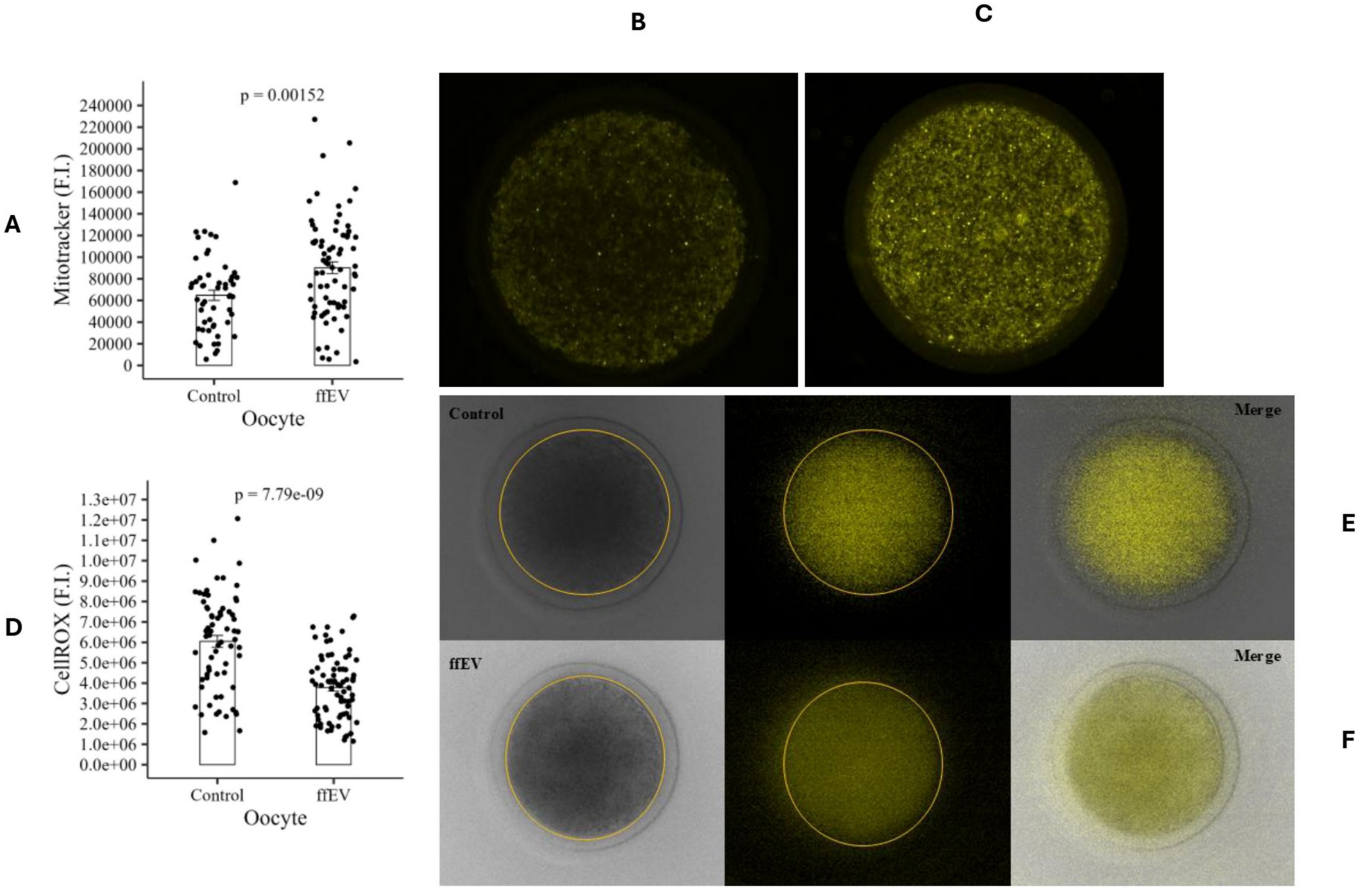
Mitochondrial activity and ROS levels analysis in bovine oocytes after IVM. **(A)** Mitochondrial membrane potential. **(B)** Control group and **(C)** ffEV group representative images of oocytes stained with MitoTracker Orange CMTMRos. **(D)** ROS levels after IVM. **(E)** Control group and **(F)** ffEV group representative oocyte stained with CellROX Orange and fluorescence merged with bright-field image. Data are presented as mean ± SEM. FI, Fluorescence intensity.

**Figure 5:**
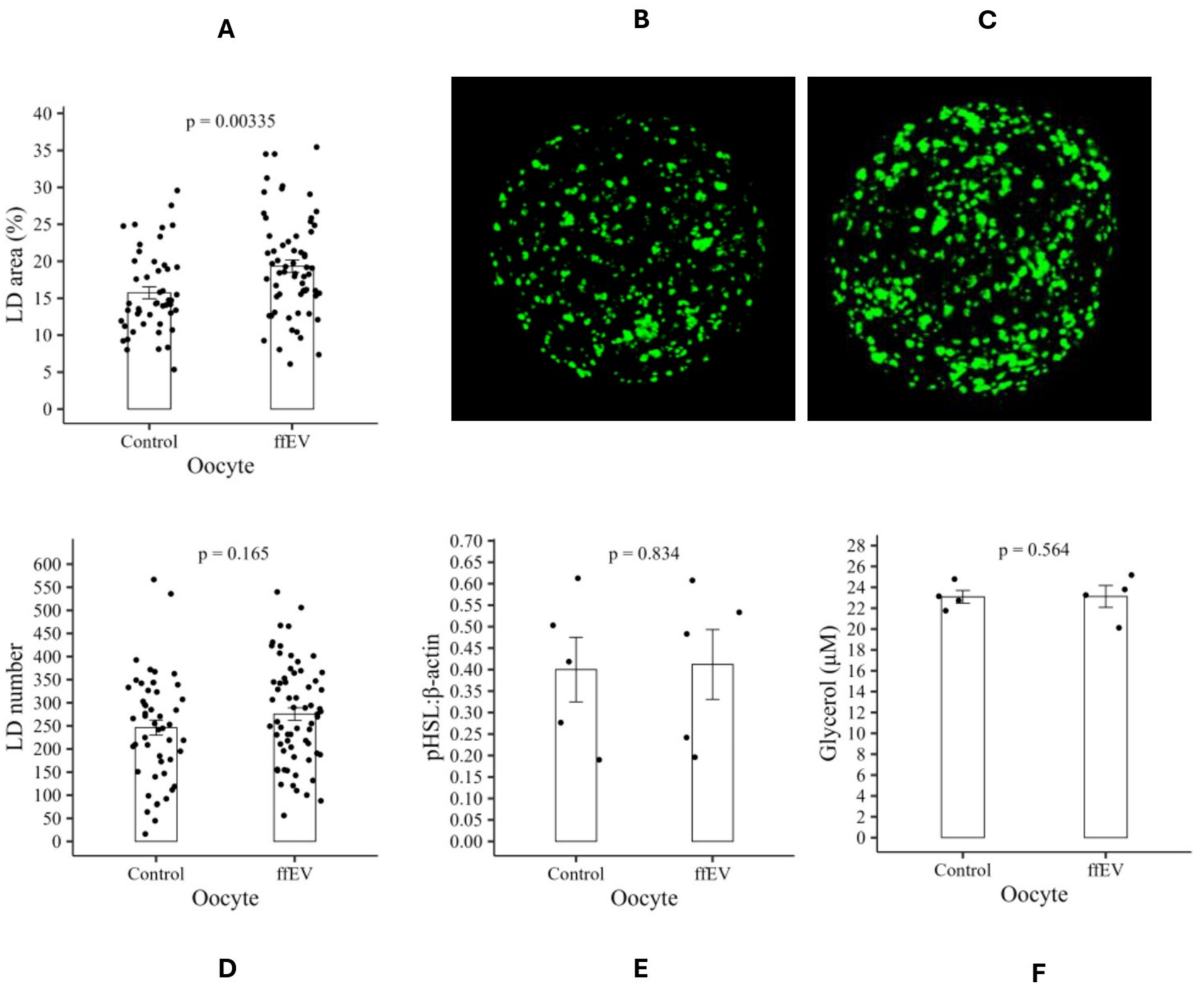
Lipid metabolism analysis in bovine oocytes after IVM. **(A)** Lipid droplet area rate. **(B)** Control group and **(C)** ffEV group representative image of oocyte stained with Bodipy 493/503. **(D)** Lipid droplet number. **(E)** Densitometry measures of pHSL bands relative to *β*-actin and **(F)** Glycerol levels in IVM media, to assess lipolytic activity. Data are presented as mean ± SEM. FI, Fluorescence intensity.

Our first experiment assessed the effect of ffEVs on bovine oocyte competence. For this, two groups were evaluated: Control (dFBS; *n* = 251 COCs) and ffEVs (dFBS supplemented with ffEV; *n* = 221 COCs). Nuclear maturation, indicated by PBE, was assessed after IVM (Table 1). PBE rates were 72.9% (Control) and 76.3% (ffEV), showing no statistically significant difference between treatments (*p* > 0.05). Mitochondrial activity, measured in control (64,783 ± 4736FI, *n* = 53, Figure 4B) and ffEVs-treated (90,114 ± 5436FI, *n* = 73, Figure 4C) oocytes, was significantly higher in the ffEVs group (*p* < 0.05, Figure 4A). Given the close association between oxidative metabolism and the generation of ROS as by-products of mitochondrial activity in oocytes, and their established link to reduced fertilization and embryo survival rates (53), we evaluated intracellular ROS levels as a complementary measure of metabolic balance. Quantified in 143 oocytes after IVM, significantly lower ROS levels (*p* < 0.05, Figure 4D) were detected in oocytes cultured with ffEVs (3,779,912 ± 180345FI, *n* = 76, Figure 4F) compared to control (6,046,772 ± 291,195, *n* = 67, Figure 4E), suggesting ffEVs may contribute to oocyte redox homeostasis by reducing ROS production or enhancing antioxidant defenses. To investigate ffEVs influence on oocyte lipid metabolism, lipid accumulation was quantified. Oocytes exposed to ffEVs (*n* = 64, Figure 5C) exhibited significantly greater LD area rate (19.3 ± 0.8%, Figure 5A, *p* < 0.05) compared to controls (15.7 ± 0.8%, *n* = 48, Figure 5B). To characterize ffEVs influence on lipid storage, LD number was evaluated (Figure 4D). While LD area rate differed, the number of individual LDs was not significantly altered (*p* > 0.05) between ffEVs-treated oocytes (276 ± 16.3LD, *n* = 64) and controls (246 ± 16.3LD, *n* = 50). As lipid storage results from the balance between synthesis and degradation (54), and given the change in oocyte lipid stores, ffEV influence on lipolysis was investigated. To assess ffEVs modulation of bovine oocyte lipolytic activity, pHSL phosphorylation, a key triglyceride hydrolysis enzyme (55), was quantified. pHSL protein expression (Figure 5E; Supplementary Figure 5) revealed no significant difference (*p* > 0.05) between control (0.40 ± 0.07 pHSL:*β*-actin rate) and ffEVs-treated (0.41 ± 0.08 pHSL:β-actin rate) groups. As a complementary approach to evaluating lipolytic activity, free glycerol concentration, a byproduct of triglyceride breakdown, was measured in the IVM medium at the end of culture. Glycerol levels were comparable between control (23.1 ± 0.6 μM) and ffEVs-treated (23.1 ± 1.0 μM) groups, with no statistically significant difference detected (*p* > 0.05; Figure 5F).

**Table 1:** Proportion of bovine oocytes undergoing first polar body extrusion (PBE) and subsequent embryo development following IVM with or without ffEVs.

Group	*n*	PBE *n* (%)	*n*	D4 *n* (%)	D7 *n* (%)
Control	251	183 (72.9)	357	212 (60.8)	140 (40.3)
ffEV	221	169 (76.3)	364	215 (59.5)	147 (40.7)

Cleavage rate (D4) is defined as the proportion of cultured oocytes reaching 8 or more cells by day 4. Blastocyst rate (D7) is defined as the proportion of cultured oocytes reaching the blastocyst stage by day 7. Both embryo development rates (D4 and D7) are calculated as the ratio of developed structures to the total number of oocytes cultured. Results presented as mean %.

### 3.4 Effect of small extracellular vesicles during IVM on competence for development and blastocyst quality

**Figure 6:**
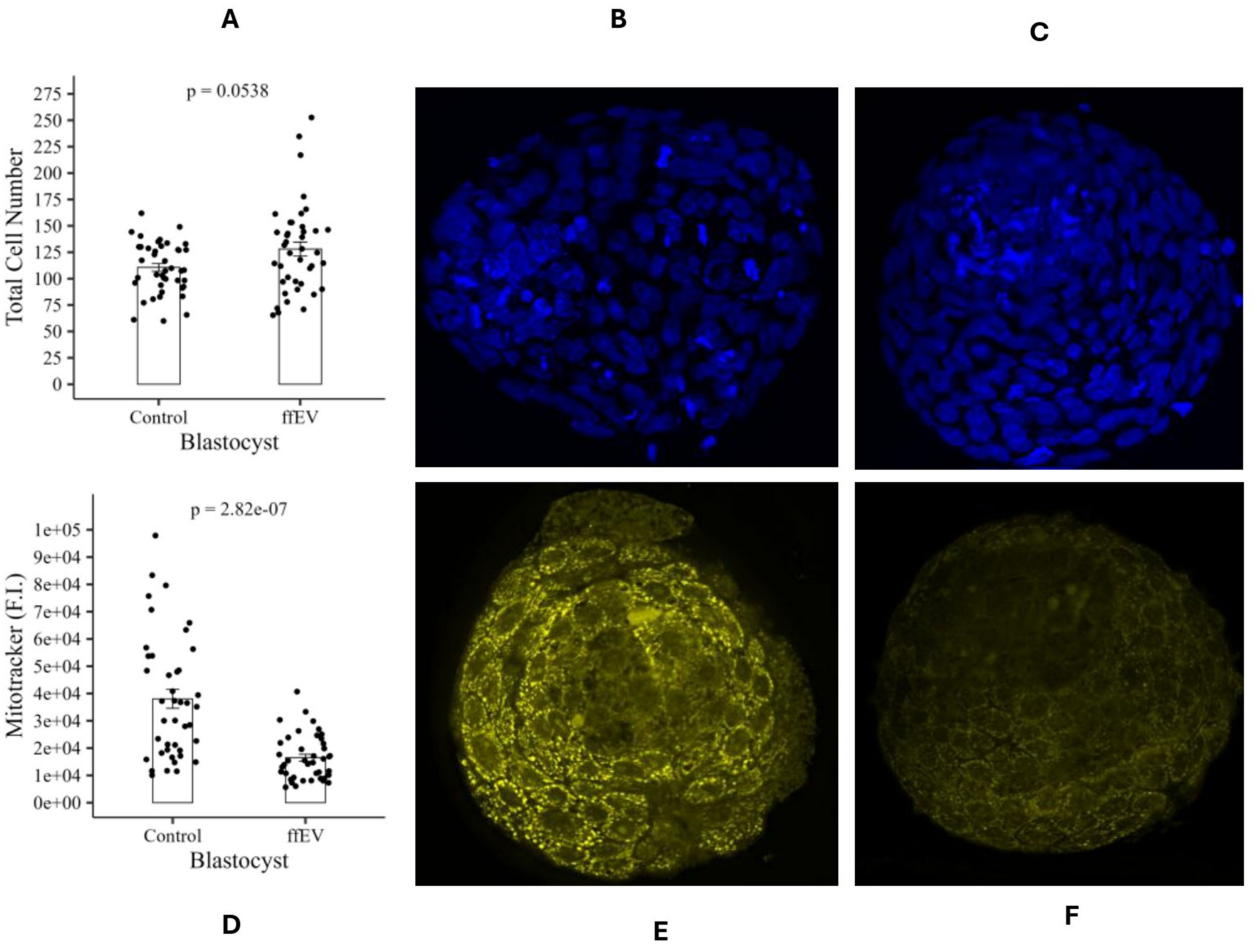
Embryo development and mitochondrial activity on day 7 of IVC. **(A)** Total cell numbers to assess embryo quality. **(B)** Mitochondrial membrane potential, to assess mitochondrial activity. **(C)** Control group and **(D)** ffEV group representative blastocyst stained with Hoechst 33342. **(E)** Control group and **(F)** ffEV group representative blastocyst stained with MitoTracker Orange CMTMRos.

**Figure 7:**
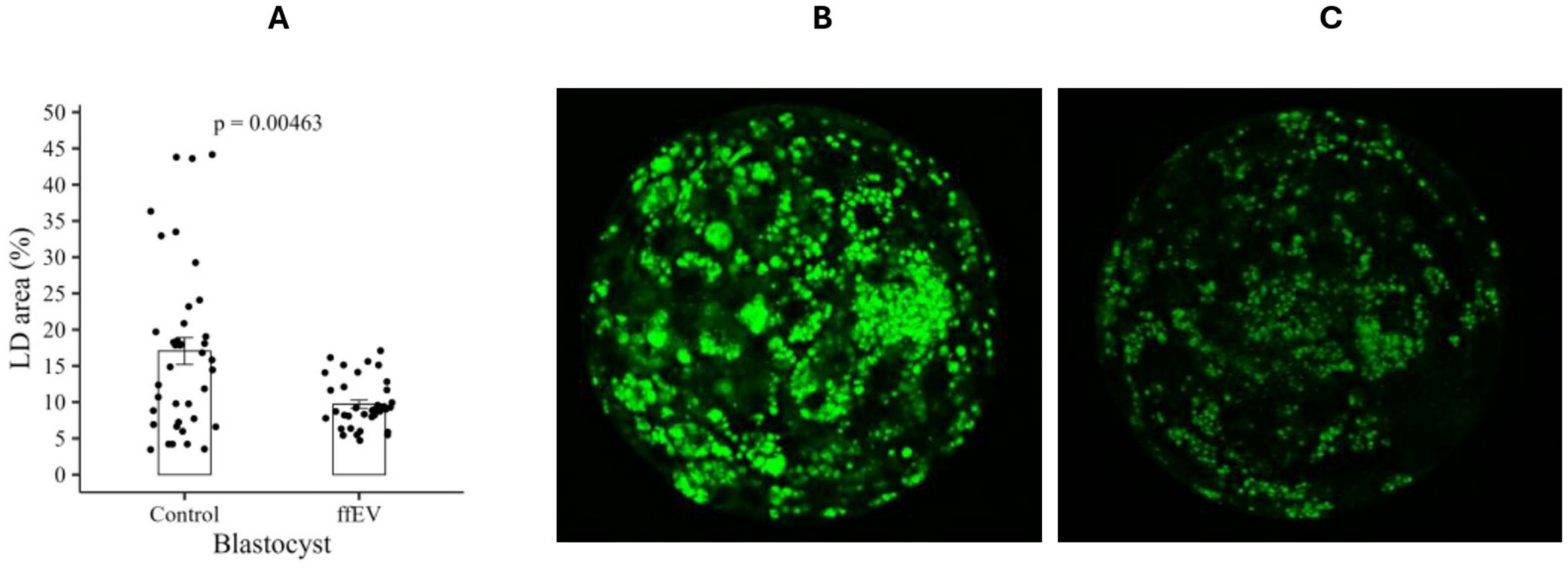
Embryo lipid metabolism on day 7 of IVC. **(A)** Lipid droplet area rate to assess lipid content. **(B)** Control group and **(C)** ffEV group representative blastocyst stained with Bodipy 493/503.

Following ffEVs supplementation during IVM, subsequent embryo development and lipid metabolism were investigated. Conventional *in vitro* culture with FBS did not significantly alter embryonic development rates (Table 1). Specifically, no differences were observed in Day 4 cleavage rates (control: 60.8%; ffEVs: 59.5%) or Day 7 blastocyst formation rates (control: 40.3%; ffEV: 40.7%) between ffEV-treated and control groups (*p* > 0.05). To assess embryonic quality, TCN was determined on day 7 blastocysts. The analysis revealed no significant differences (*p* > 0.05; Figure 6A) between control (111 ± 3.70 counts, *n* = 43, Figure 6B) and ffEV-treated (128 ± 6.65 counts, *n* = 41, Figure 6C) embryos. Mitochondrial activity, a key indicator of metabolic competence, was assessed in Day 7 blastocysts using a fluorescent dye-based assay. In mitochondrial activity was observed significant decrease (*p* < 0.05, Figure 6D) in embryos derived from ffEVs-treated (16,448 ± 1281FI, *n* = 42, Figure 6F) versus control (38,045 ± 3462FI, *n* = 42, Figure 6E). To assess ffEV modulation of embryo lipid metabolism, Day 7 blastocyst LD content was quantified. The ffEV-treated embryos (9.72 ± 0.5%, *n* = 36, Figure 7C) showed significantly reduced lipid accumulation (*p* < 0.05; Figure 7A) compared to controls (17.1 ± 1.8%, *n* = 39, Figure 7B).

### 3.5 Expression of RNA (mRNA and miRNA) in oocytes and cumulus cells matured in the presence or absence of small extracellular vesicles, and comparison with RNA contained in small extracellular vesicles

To investigate potential molecular mediators of observed phenotypic effects from ffEV supplementation, a comprehensive panel of transcripts linked to lipid metabolism (35 mRNAs, 41 miRNAs) and antioxidant function (4 mRNAs) was analyzed (Supplementary Table 1). The presence of several of these transcripts within ffEVs was confirmed (pool of 10 biological replicates), and their expression levels were assessed in oocytes and CCs following IVM with ffEV supplementation. In oocytes (4 biological replicates), 16 lipid metabolism and all four antioxidant enzyme transcripts were detected (Table 2), while in CCs (4 biological replicates), 15 lipid metabolism and three of the four antioxidant enzyme transcripts (Table 3). Oocyte transcripts primarily included antioxidant enzymes (*SOD2, SOD1, GPX4*) and the lipid intracellular transport protein (*FABP3*, Supplementary Figure 2A). CC predominantly expressed lipid metabolism genes (*PLIN2* and *FABP3*), and antioxidant enzymes (*GPX1, GPX4,* and *SOD1*, Supplementary Figure 2B). The ffEVs were enriched in lipid metabolism genes (*ELOVL5* and *LDLR*) and antioxidant transcripts (*SOD2* and *GPX1*, Supplementary Figure 2C) (Table 4).

**Table 2:** Relative abundance of mRNA transcripts in oocytes.

Gene	Control	ffEV	*p*-value
	2^-∆Ct^±SD	2^-∆Ct^±SD	
Lipid metabolism			
ACCA	0.021±0.004	0.021±0.001	0.8857
AMPKA1	0.026±0.014	0.024±0.011	0.8857
AMPKA2	0.061±0.003	0.060±0.005	0.6857
CD36	0.041±0.011	0.036±0.011	0.8857
DGAT1	0.016±0.009	0.015±0.008	1.0000
ELOVL5	0.699±0.585	0.595±0.537	0.6857
FABP3	1.079±0.160	1.073±0.160	0.8857
FABP5	0.578±0.114	0.511±0.050	0.3429
LDLR	0.011±0.006	0.016±0.011	0.4857
LIPE	0.015±0.017	0.012±0.012	0.8857
PLIN2	0.422±0.135	0.430±0.082	0.6857
PLIN3	0.026±0.006	0.025±0.006	1.0000
PNPLA2	0.010±0.009	0.010±0.008	1.0000
PPARG	0.220±0.052	0.225±0.040	0.8857
PPARGC1A	0.018±0.016	0.014±0.010	0.8857
PPARGC1B	0.017±0.004	0.022±0.006	0.2000
Antioxidant enzymes			
GPX1	0.032±0.003	0.037±0.006	0.1143
GPX4	0.986±0.098	0.918±0.084	0.3429
SOD1	1.400±0.363	1.279±0.319	0.6857
SOD2	1.991±0.614	1.725±0.310	0.4857

**Table 3:** Relative abundance of mRNA transcripts in CC.

Gene	Control	ffEV	*p*-value
	2^-∆Ct^±SD	2^-∆Ct^±SD	
Lipid metabolism			
ACCA	0.011±0.002	0.012±0.002	0.4795
AMPKA2	0.004±0.001	0.004±0.001	0.2752
CD36	0.004±0.001	0.005±0.001	0.2752
CPT1A	0.001±0.001	0.003±0.003	0.2752
DGAT1	0.002±0.001	0.002±0.001	0.2752
FABP3	0.256±0.109	0.318±0.094	0.8273
FABP5	0.001±0.0005	0.001±0.001	0.5127
FASN	0.009±0.010	0.007±0.014	0.7728
LDLR	0.143±0.025	0.147±0.030	0.7728
LPL	0.005±0.001	0.004±0.002	0.8273
PLIN2	0.359±0.122	0.336±0.134	0.8273
PLIN3	0.012±0.001	0.013±0.002	0.5637
PPARG	0.050±0.008	0.055±0.010	0.2888
PPARGC1A	0.0001±0.0001	0.00003±0.00001	0.2752
PPARGC1B	0.0004±0.00003	0.0004±0.0002	0.5637
Antioxidant enzymes			
GPX1	0.277±0.069	0.258±0.110	0.5637
GPX4	0.223±0.020	0.205±0.093	1.0000
SOD1	0.184±0.038	0.212±0.036	0.2482

**Table 4:** Relative abundance of mRNA transcripts detected in ffEV.

Function	Target	2^-∆Ct^±SD
Fatty acid synthesis	ELOVL5	0.31±0.008
Regulation of access to lipolytic enzymes	PLIN2	0.14±0.027
Lipogenesis	ACCA	0.07±0.021
	AMPKA2	0.14±0.012
	PPARGC1A	0.01±0.003
	PPARG	0.004±0.002
Lipid uptake and transport	LPL	0.04±0.002
	FABP5	0.10±0.013
	CD36	0.001±0.0003
Cholesterol receptor	LDLR	0.30±0.040
Antioxidant	GPX1	0.18±0.015
	GPX4	0.13±0.003
	SOD1	0.14±0.002
	SOD2	0.19±0.024

To assess the potential contribution of miRNAs to ffEVs supplementation effects, the expression of 41 candidate miRNAs involved in lipid metabolism and related pathways was analyzed in oocytes, CC and ffEVs. In oocytes, only 11 of the 41 selected miRNAs were detected and their expression remained unchanged following ffEVs supplementation (*p* > 0.05, Table 5). For CC, 40 miRNAs were detected post-IVM across treatments (Table 6). Notably, bta-miR-23b-3p was significantly less abundant in the ffEVs-treated group (*p* < 0.05, Figure 2), representing the sole miRNA exhibiting a treatment-related effect.

**Table 5:** Relative abundance of miRNA transcripts in oocytes.

miRNA	Control	ffEV	*p*-value
	2^-∆Ct^±SD	2^-∆Ct^±SD	
bta-miR-122	0.044±0.048	0.039±0.020	0.7728
bta-miR-150	0.019±0.004	0.037±0.028	1.0000
bta-miR-155	0.505±0.310	0.573±0.094	0.5637
bta-miR-18a	0.253±0.166	0.268±0.033	0.3865
bta-miR-18b	0.059±0.021	0.081±0.023	0.3865
bta-miR-194	0.029±0.012	0.036±0.016	0.5637
bta-miR-23b-3p	1.791±0.313	2.990±1.444	0.2482
bta-miR-24-3p	0.105±0.047	0.152±0.039	0.2482
bta-miR-27a-3p	0.050±0.013	0.054±0.023	0.5637
bta-miR-652	0.052±0.006	0.096±0.048	0.2482
bta-miR-758	0.027±0.014	0.019±0.008	0.2482

**Table 6:** Relative abundance of miRNA transcripts in CC.

miRNA	Control	ffEV	*p*-value
	2^-∆Ct^±SD	2^-∆Ct^±SD	
bta-miR-107	0.133±0.064	0.137±0.026	0.386
bta-miR-1193	0.039±0.038	0.033±0.031	0.827
bta-miR-122	0.021±0.025	0.017±0.018	0.773
bta-miR-1298	0.027±0.032	0.038±0.037	0.480
bta-miR-133b	0.033±0.035	0.030±0.033	0.773
bta-miR-140	1.163±0.385	1.087±0.290	0.564
bta-miR-147	0.022±0.021	0.020±0.017	0.773
bta-miR-150	0.043±0.043	0.025±0.029	0.480
bta-miR-181b	0.782±0.414	0.793±0.066	0.480
bta-miR-188	0.230±0.011	0.162±0.128	1.000
bta-miR-18a	1.316±0.337	1.105±0.217	0.386
bta-miR-18b	0.356±0.132	0.309±0.057	1.000
bta-miR-192	0.085±0.040	0.074±0.021	0.564
bta-mir-193a	0.021±0.023	0.020±0.021	0.827
bta-miR-194	0.353±0.178	0.288±0.132	0.386
bta-miR-196b	0.040±0.038	0.030±0.021	1.000
bta-miR-199a-5p	0.158±0.066	0.124±0.002	0.289
bta-miR-199b	0.024±0.027	0.020±0.023	0.773
bta-miR-224	0.218±0.089	0.220±0.081	0.773
bta-miR-23b-3p	1.018±0.311	0.638±0.067	0.043
bta-miR-24-3p	5.279±2.001	4.531±2.104	0.564
bta-miR-27a-3p	5.576±2.223	5.024±2.807	0.564
bta-miR-3064	0.054±0.039	0.051±0.050	0.773
bta-miR-329b	0.030±0.037	0.036±0.031	0.724
bta-miR-345-3p	0.031±0.035	0.029±0.020	1.000
bta-miR-379	0.033±0.021	0.027±0.018	0.773
bta-miR-380-3p	0.030±0.032	0.024±0.023	0.564
bta-miR-410	0.026±0.029	0.023±0.024	0.773
bta-miR-412	0.028±0.030	0.019±0.026	0.480
bta-miR-448	0.045±0.052	0.043±0.057	1.000
bta-miR-455-5p	0.044±0.031	0.036±0.023	1.000
bta-miR-487a	0.025±0.030	0.023±0.027	0.564
bta-miR-562	0.038±0.045	0.033±0.038	0.564
bta-miR-652	1.112±0.372	1.043±0.364	0.773
bta-miR-677	1.371±0.851	1.447±0.192	1.000
bta-miR-708	0.682±0.330	0.588±0.148	0.773
bta-miR-758	0.067±0.055	0.060±0.065	1.000
bta-miR-873	0.041±0.032	0.031±0.021	1.000
bta-miR-875	0.048±0.038	0.042±0.036	0.773
bta-miR-96	0.510±0.267	0.391±0.283	0.564

Analysis of transcripts in ffEVs identified 28 of 41 studied miRNAs (Table 7), indicating a diverse cargo potentially capable of influencing recipient cells. Oocytes exhibited high expression of bta-miR-23b-3p, bta-miR-155, and bta-miR-18a (Supplementary Figure 3A). In CC, bta-miR-27a-3p, bta-miR-677, and bta-miR-140 were predominant (Supplementary Figure 3B). ffEVs were enriched in bta-miR-24-3p, bta-miR-27a-3p, and bta-miR-23b-3p, which are consistently associated with lipid metabolism and follicular function (Supplementary Figure 3C). ffEVs supplementation did not substantially alter oocyte miRNA profiles, and only minimally affected CC.

**Table 7:** Relative abundance of miRNA transcripts detected in ffEV.

microRNA	2^-∆Ct^±SD
bta-miR-107	0.0057±0.0003
bta-miR-133b	0.0015±0.0013
bta-miR-140	0.1365±0.0098
bta-miR-147	0.0007±0.0000
bta-miR-150	0.0064±0.0025
bta-miR-181b	0.0125±0.0011
bta-miR-188	0.0065±0.0011
bta-miR-18a	0.0709±0.0040
bta-miR-18b	0.0352±0.0020
bta-miR-192	0.0089±0.0010
bta-miR-194	0.0051±0.0001
bta-miR-199a-5p	0.0665±0.0024
bta-miR-199b	0.0291±0.0019
bta-miR-224	0.0137±0.0021
bta-miR-23b-3p	0.1705±0.0057
bta-miR-24-3p	0.4734±0.0138
bta-miR-27a-3p	0.2586±0.0225
bta-miR-345-3p	0.0012±0.0000
bta-miR-380-3p	0.0038±0.0019
bta-miR-410	0.0011±0.0005
bta-miR-455-5p	0.0102±0.0012
bta-miR-487a	0.0003±0.0001
bta-miR-562	0.0002±0.0001
bta-miR-652	0.0482±0.0045
bta-miR-677	0.0026±0.0001
bta-miR-708	0.0021±0.0002
bta-miR-758	0.0009±0.0007
bta-miR-873	0.0063±0.0005
bta-miR-875	0.0006±0.0000

## 4 Discussion

IVM systems often fail to replicate the follicular microenvironment, leading to reduced quality, altered lipid metabolism, and redox imbalance in oocytes and resulting embryos. Despite EVs being abundant in reproductive fluids and known to transfer biomolecules such as RNAs and proteins, their precise role in modulating oocyte and *in vitro*-produced embryo lipid metabolism remains unclear. In the present study, we investigated the outcomes of supplementing ffEVs to the IVM medium on oocyte maturation, developmental competence, and metabolism-related parameters. Our study revealed that ffEVs modulated oocyte physiology by increasing lipid accumulation, enhancing mitochondrial activity, and decreasing intracellular ROS levels. These metabolic shifts during maturation were linked to altered embryo lipid content and mitochondrial activity, suggesting long-lasting effects of ffEVs exposure during IVM on early embryonic metabolic programming.

Nuclear maturation was not enhanced by ffEVs supplementation, similar to the findings of other studies (19, 56–59). In contrast, Hung et al. (16) observed increased CC expansion, as an indicator of maturation, and Pakniyat et al. (23) reported higher metaphase II rates (from 75 to 80%). Similarly, a lack of effect of ffEV on subsequent embryo development was also observed in this study, as well as in others (21, 57, 60, 61), but increased development, has also been described (20, 27, 58, 59). It may be worth considering that positive effects of ffEVs may be more evident in challenging environments. Under heat stress (21), aging (62), metabolic stress (57) or vitrification (58), ffEVs during IVM improved embryo development and/or maturation, exerting a protective effect on the oocyte. Nevertheless, the variable results regarding the use of ffEV during IVM on maturation and development rates (no effect or increase) may be attributed to varying conditions between studies. A single study reported an increase in maturation rates (23). In this study, ffEVs were included during the first 18 h IVM (next 4.5 h without), EVs were obtained from FF collected from preovulatory follicles of synchronized Holstein heifers by OPU, and isolated by using a precipitation kit. In our study, FF was collected from 3–6 mm follicles of abattoir ovaries, isolated by SEC and added for the entire IVM period. Regarding embryo development, Singina et al. (59) used OPU-collected COCs for IVM and we used abattoir-derived COCs, and isolation method (UC). Esposito et al. (27) used a different IVM system (2 h prematuration with cAMP elevating agents followed by 22 h IVM) and ffEVs were isolated by UC, but from FF which contained oocytes that resulted in blastocysts (retrospective study). Different isolation methods have been shown to influence outcomes on embryo development (22, 60). Another aspect to consider, which is a limitation of our study, is the concentration used, as we have not tested other concentrations, but chose one based on a previous study (20). However, only two studies have tested different concentrations. Esposito et al. (27) evaluated 1× 10^6^, 1×10^7^ and 1×10^8^ particles/mL and found improved blastocyst development with 1×10^7^ particles/mL. Paknyiat et al. (23) mention testing different concentrations, but the results were not presented and they used 40 μg/mL. Published work varies substantially in terms of concentration used and how it is informed: 10% concentration of FF (19, 20, 56); from 12.5 to 200 μg/mL: (16, 22); 1×10^7^ to 1.6× 10^10^ particles/mL (21, 27). As there are no standardized parameters for isolation and supplementation of ffEVs, all these studies present substantial differences in several steps among themselves and compared to ours. Therefore, much remains to be studied and improved regarding the use of ffEVs for IVM culture.

In addition to oocyte maturation and competence, ffEVs may play a role in modulating oocyte quality by influencing cytoplasmic maturation. In our study, oocytes treated with ffEVs demonstrated higher mitochondrial activity and lower levels of ROS than controls. These oocytes also exhibited increased lipid accumulation. These observations suggest that the metabolic state of the oocyte may be modulated by intercellular communication within the follicular environment, likely mediated by EVs, and may be partially mimicked during *in vitro* culture with their supplementation. Besides, embryo lipid content and mitochondrial activity were also modulated, showing the later effects on the resulting embryo metabolism. The elevated mitochondrial activity detected in oocytes indicates that ffEVs can improve oocyte quality. This enhancement is significant as mitochondria supply ATP for energy-intensive meiotic processes (30). Although specific bovine studies remain limited, reports on porcine oocytes show these vesicles modulate mitochondrial membrane potential and promote antioxidant defense mechanisms (63–65). In bovine oocytes, Pakniyat et al. (23) demonstrated that ffEVs and ampullary oviductal fluid-derived EVs enhance mitochondrial quantity further supporting improved mitochondrial function in oocytes by ffEVs. How EVs modulate mitochondrial function in oocytes is yet unknown. One mechanism could involve cyclic adenosine monophosphate (cAMP) regulation, as ffEVs increase transcription of enzymes responsible for cAMP synthesis in oocytes (61), and elevated intracellular cAMP levels in bovine oocytes enhance ATP content and mitochondrial activity (46). Proteomic analysis of bovine ffEVs identified several mitochondrial proteins (18). This implies that these elements may be transferred to COCs via ffEVs, affecting mitochondrial activity and promoting oocyte maturation.

Mitochondria generate ROS and act as central regulators of cellular redox balance (31). Maintaining mitochondrial function ensures this equilibrium (41), but high ROS levels can oxidize lipids, proteins, and DNA, resulting in cellular damage (66). In oocytes, this oxidative stress compromises quality by inducing mitochondrial dysfunction (67), and higher ROS levels are associated with impaired oocyte developmental competence (53, 68, 69). We found that ROS levels were lower in bovine oocytes supplemented with ffEVs, suggesting that ffEVs may mitigate *in vitro* redox imbalance counteracting apoptosis and premature aging that may occur in vitro (70). This is the first study to demonstrate this impact on bovine oocytes, and the results are consistent with earlier research conducted in pigs by Kim et al. (63). These investigators noted that oocytes supplemented with porcine ffEVs had lower ROS levels.

Protective molecules carried by ffEVs may exert antioxidant effects upon delivery. According to Uzbekova et al. (18), their cargo contains antioxidant proteins that could potentially protect cells from oxidative stress. In pigs, Kim et al. (63) observed that ffEVs could increase transcripts for the antioxidant catalase and the levels of the non-enzymatic antioxidant glutathione. Therefore, our data, along with other studies, demonstrate that ROS levels in ffEVs-treated oocytes may be significantly reduced. This supports the potential of ffEVs to improve in vitro oocyte quality by confirming their antioxidant role in oocyte protection. Although mRNA for four selected antioxidants were detected in ffEVs, their levels in oocytes and cumulus cells was not affected by ffEVs, therefore, other antioxidants and/or molecules within the ffEVs could contribute to the decrease in ROS levels observed in this study. The overlapping “fateful triad” of mitochondrial activity, ROS levels, and lipid content affects oocyte quality and the developmental potential of bovine embryos (39).

Reduced embryo cryotolerance is generally associated with cytoplasmic lipid accumulation (71). In the present study, oocytes incubated with ffEVs exhibited higher lipid content, but accompanied by decreased oxidative stress, and increased mitochondrial activity. This suggests that ffEVs may advantageously control lipid accumulation in vitro, in contrast to normal in vitro maturation, where lipid accumulation is frequently associated with mitochondrial dysfunction (72, 73). Monounsaturated fatty acids, such as oleic acid, are regarded as non-toxic and can counteract the lipotoxic effects of saturated fatty acids by facilitating lipid storage and augmenting *β*-oxidation (33, 74). Pakniyat et al. (23) recently reported that blastocysts derived from oocytes matured with ffEVs showed reduced presence of detrimental saturated fatty acid species. Thus, ffEVs may mitigate cellular stress by either supplying or stimulating the synthesis/storage of advantageous lipids, thereby modulating the balance between detrimental and protective lipid species, potentially through the sequestration of fatty acids to prevent their cytoplasmic accumulation at toxic concentrations (75). This possibility should be addressed in future studies.

Lipid accumulation in COCs during IVM may be associated with decreased levels of β-oxidation (35), resulting in lower utilization of fatty acids for energy production. Therefore, lipolysis indicators were analyzed. HSL plays a central role in the hydrolysis of triglycerides stored in LD (76). Its activation, by phosphorylation (pHSL), occurs during maturation and is more abundant in oocytes than in CC (77, 78). The oocytes from the control and ffEV groups did not differ in pHSL levels. Leal et al. (29) reported an increase in pHSL associated with reduced lipid accumulation in embryos exposed to uterine and oviductal EVs. However, the ffEVs in our study had a different effect. Glycerol levels in the IVM medium, a byproduct of triglyceride hydrolysis suggestive of lipase activity (77, 79), were also assessed as an indirect indicator of lypolisis in the whole COCs. Again, there was no difference between groups. As cellular lipid content is the consequence of the balance between synthesis and hydrolysis (80), similar pHSL and glycerol levels imply that the higher lipid content in oocytes treated with ffEVs is probably caused by increased lipogenesis and/or lipid uptake rather than decreased lipid breakdown.

Given the higher mitochondrial activity and lipid content in oocytes exposed to ffEVs during IVM, this study investigated whether similar patterns occurred in the resulting embryos. Embryo quality was also evaluated in terms of TCN. Total cell numbers were not affected by ffEVs, similar to findings of previous studies (23, 50, 53, 54). An increase in TCN was reported by Asaadi et al. (22) and Pérez-García et al. (60), but these differences among studies are probably due to the varying methodological procedures. In contrast, the role of ffEVs in the metabolic modulation of oocytes and subsequent embryos was evident, as lipid content was reduced in the resulting blastocysts, pointing to an improved quality of these embryos. Previous studies have indicated that IVM conditions that affect the lipid metabolism of oocytes may affect the lipid contents of resulting embryos with increase in oocyte lipids leading to decrease in embryos and vice-versa (81–83). These findings are suggestive that embryo lipid metabolism adapts to that of the originating oocyte. Anyway, as excessive lipid accumulation in IVP embryos directly impairs cryopreservation, resulting in reduced pregnancy rates (39), IVM with ffEVs lowering the lipid contents in resulting embryos, could possibly improve their cryotolerance. Supplementation of oviductal and uterine EVs during IVC lead to embryos that had lower lipid contents and higher cryotolerance (29), so a similar outcome could be achieved using ffEVs during IVM. This possibility remains to be addressed. Curiously, while lipids decreased in embryos, mitochondrial activity was also lower. Although we did not measure ROS in blastocysts, the decreased mitochondrial activity in embryos could be related to the reduced oxidative stress during maturation maintained through early development. ROS levels are controlled by mitochondria, and embryo viability depends on the maintenance of an ideal membrane potential (84, 85).

ffEVs transport bioactive molecules, such as miRNAs and mRNAs, that control recipient cell activity (19, 20). As both oocytes (57) and CC internalize ffEVs (19), selected mRNAs and miRNAs associated with lipid metabolism were examined in ffEVs, as well as in CC and oocytes, to investigate their contribution to the observed outcomes. In our experiment, the incubation of ffEVs during IVM produced phenotypic effects in oocytes, but neither the expression levels of the selected mRNAs in oocytes or CC nor the miRNAs in oocytes showed significant changes. In CC, only one of the miRNAs (miR-23b-3p) had decreased expression in the ffEVs group. Although other studies have shown that ffEVs can modify mRNAs or miRNAs in cells that uptake them (19, 20), this was not the case in this study. Our results indicate that ffEVs may influence lipid metabolism and redox balance through other mRNAs and/or miRNAs that were not among those selected for the experiments. Besides, EVs supplementation can also alter oocyte phenotypes through proteins (86, 87) and lipids (57, 88), so the specific mechanism by which EVs cargoes regulated oocyte metabolism warrants further investigation.

While the present study showed only changes in bta-miR-23b-3p expression following ffEV supplementation, emerging evidence supports a role for this miRNA in modulating mitochondrial metabolism. The downregulation of bta-miR-23b-3p in CC following ffEV treatment may de-repress several predicted targets encoding mitochondrial proteins, allowing target mRNAs to be translated more efficiently without requiring transcriptional upregulation (89).

miR-23b-3p is not only predicted to target metabolic genes but has also been experimentally validated as regulator of energy metabolism, mitochondrial function, and lipid metabolism in different biological contexts. This microRNA appears to act as a “metabolic switch,” capable of suppressing mitochondrial respiration (90), modulating lipid synthesis and accumulation via *PPARGC1B* and other lipogenic genes (91), and promoting metabolic phenotypes associated with aging and inflammation (92).

Interventions that promote metabolic improvements related with mitochondrial activity and ROS balance and regulation of lipids, through supplementation with ffEVs, suggest an improvement in cytoplasmic competence and embryonic resilience, with a potentially beneficial effect on cryopreservation (38, 47, 93, 94). With implications for embryonic development, this study offers insights into how ffEVs regulate mitochondrial activity and lipid metabolism in oocytes and may later affect resulting embryos as well. In conclusion, while there was no discernible effect on nuclear maturation or embryonic development rates under the tested conditions, complex metabolic dynamics are shown by lipid metabolism and mitochondrial activity, suggesting ffEVs exposure may impact long-term embryonic metabolic programming. The selected miRNAs and mRNAs studied and present as ffEVs cargo, do not appear to be involved in the results observed, indicting other molecules may be implicated. The necessity for technical standardization is highlighted by the variability in outcomes that is associated with variations in isolation methods and culture settings. Further research is necessary to elucidate mechanisms and optimize applications, as these findings support ffEVs as modulators of the *in vitro* milieu with potential to improve oocyte and embryo quality.

## Data Availability

The original contributions presented in the study are included in the article/Supplementary material, further inquiries can be directed to the corresponding author.
