# Supplementary material for: Follicular fluid extracellular vesicles improve bovine oocyte quality via lipid and mitochondrial modulation
Source: Front Vet Sci. 2026 Jan 14;12:1703475. doi: 10.3389/fvets.2025.1703475 (PMC12849768; doi:10.3389/fvets.2025.1703475)

Supplementary Material

Supplementary Figure 1 | Hormone concentrations of (A) Progesterone (P4) and (B) Estradiol (E2) in FF samples, (C) E2:P4 ratio calculated for each sample. Data are presented as mean ± standard error of the mean (SEM).

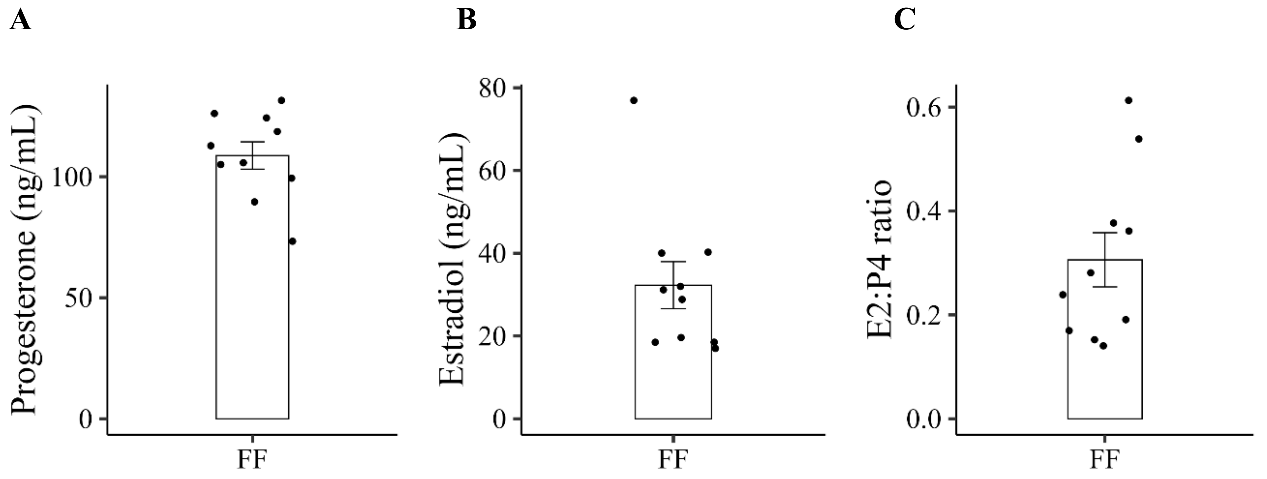

Supplementary Figure 2 | Expression levels of lipid metabolism and antioxidant-associated transcripts in (A) oocytes, (B) CC and (C) ffEV

A

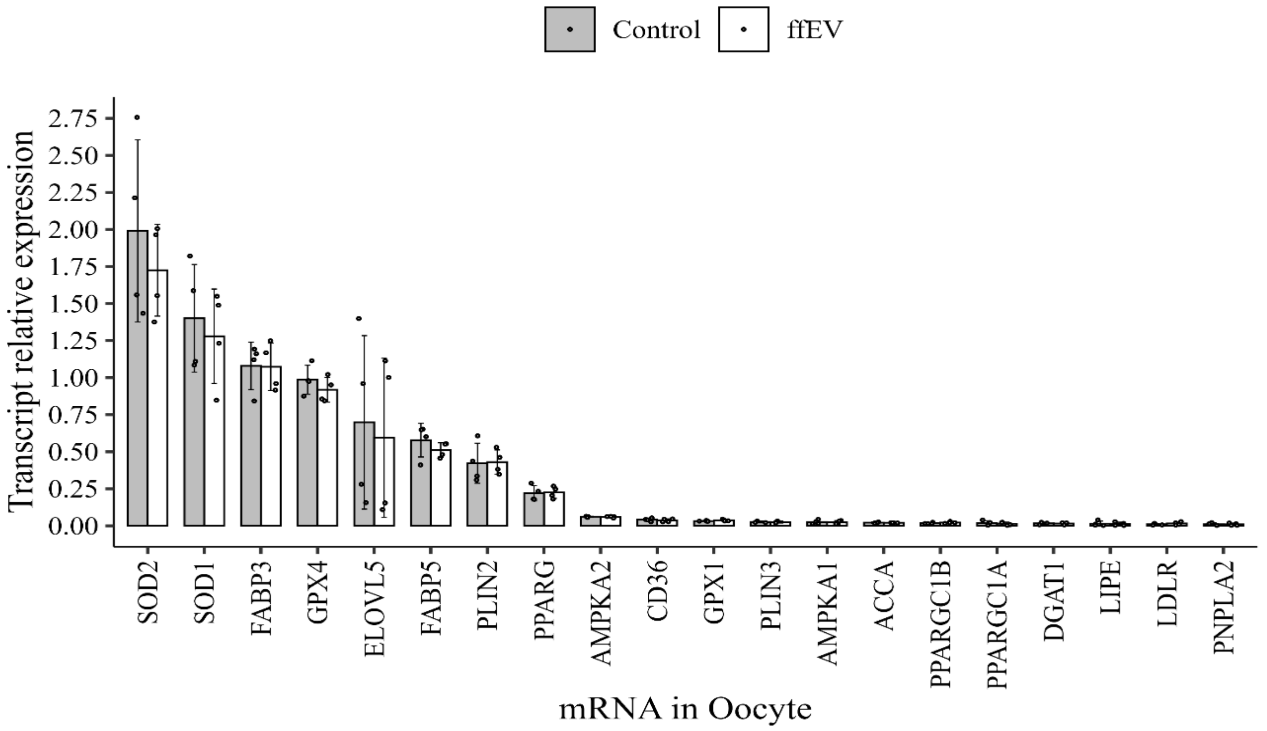

B

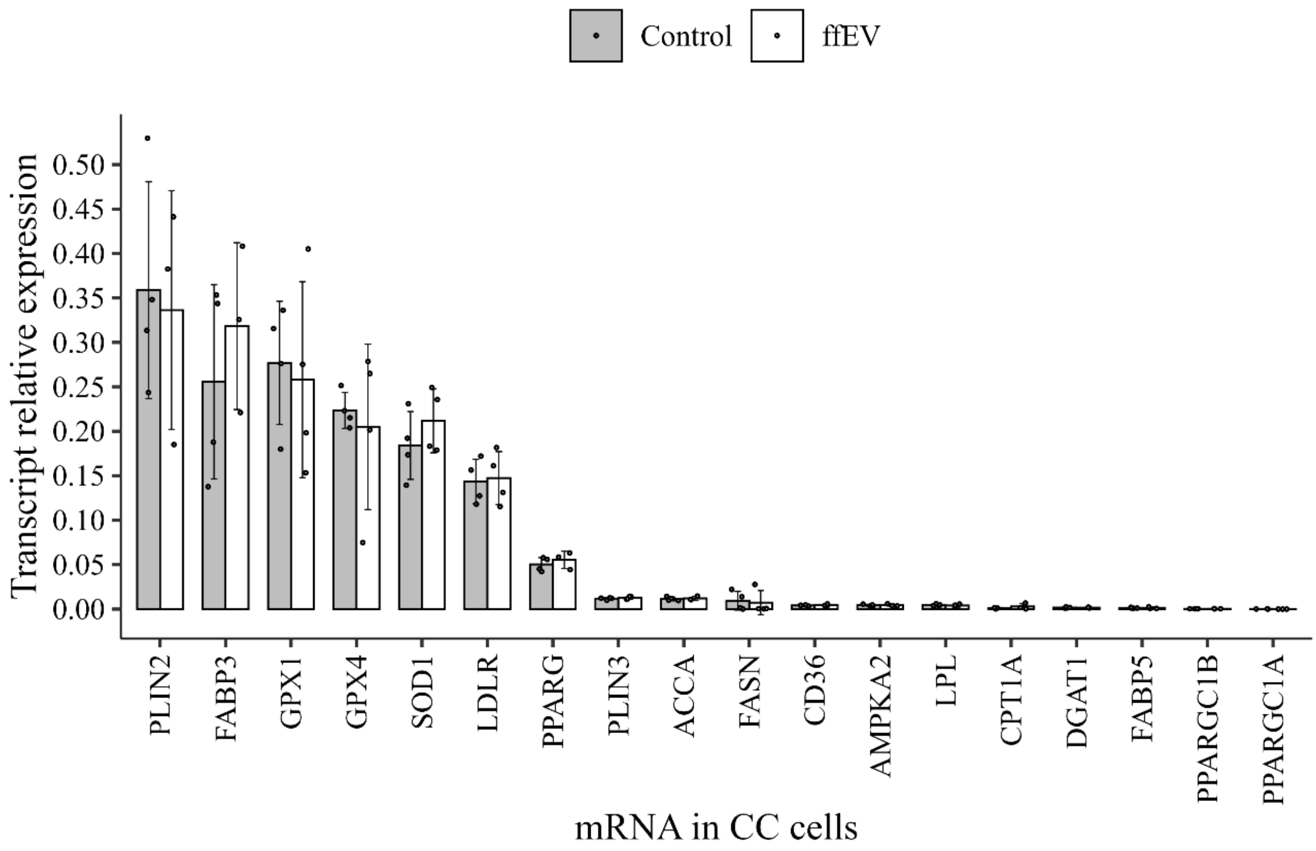
Control
ffEV
Transcript relative expression
0.50
0.45
0.40
0.35
0.30
0.25
0.20
0.15
0.10
0.05
0.00
PLIN2
FABP3
GPX1
GPX4
SOD1
LDLR
PPARG
PLIN3
ACCA
FASN
CD36
AMPKA2
LPL
CPT1A
DGAT1
FABP5
PPARGC1B
PPARGC1A
mRNA in CC cells

C

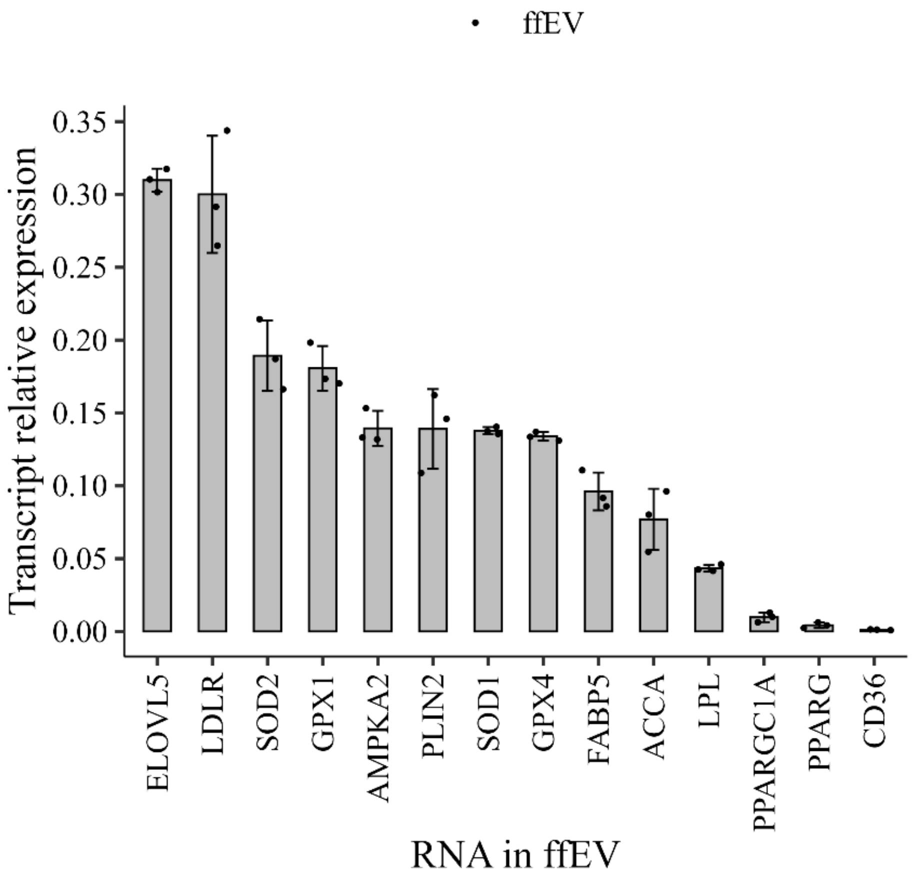
ffEV
Transcript relative expression
0.35
0.30
0.25
0.20
0.15
0.10
0.05
0.00
ELOVL5
LDLR
SOD2
GPX1
AMPKA2
PLIN2
SOD1
GPX4
FABP5
ACCA
LPL
PPARGC1A
PPARG
CD36
RNA in ffEV

Supplementary Figure 3 | Expression levels of lipid metabolism-associated miRNA transcripts in (A) oocytes, (B) CC and (C) ffEV

A

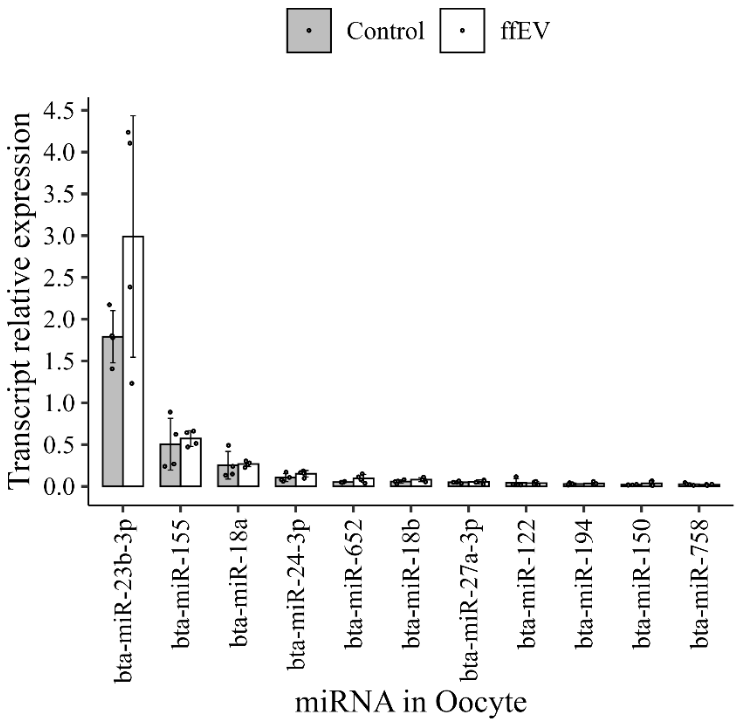

B

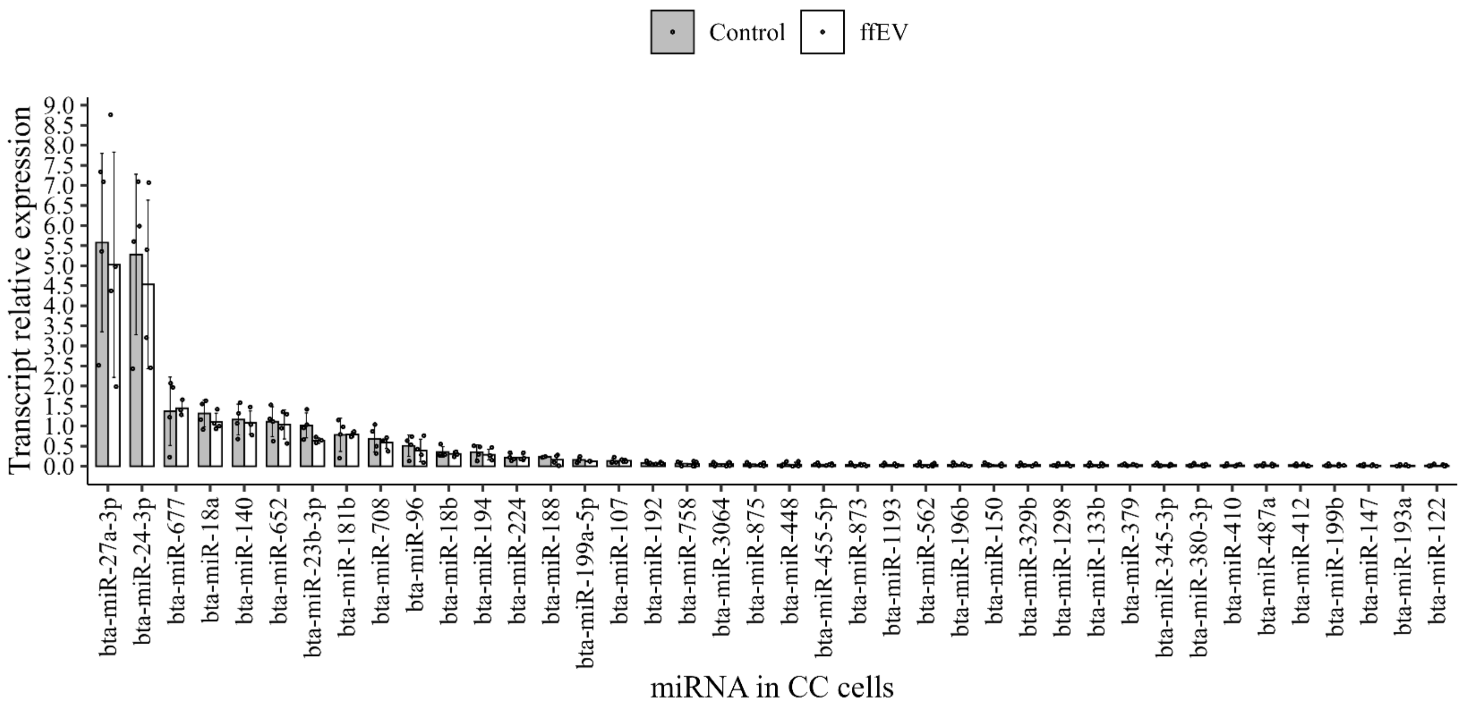
Control
ffEV
Transcript relative expression
0.0
0.5
1.0
1.5
2.0
2.5
3.0
3.5
4.0
4.5
5.0
5.5
6.0
6.5
7.0
7.5
8.0
8.5
9.0
bta-miR-27a-3p
bta-miR-24-3p
bta-miR-677
bta-miR-18a
bta-miR-140
bta-miR-652
bta-miR-23b-3p
bta-miR-181b
bta-miR-708
bta-miR-96
bta-miR-18b
bta-miR-194
bta-miR-224
bta-miR-188
bta-miR-199a-5p
bta-miR-107
bta-miR-192
bta-miR-758
bta-miR-3064
bta-miR-875
bta-miR-448
bta-miR-455-5p
bta-miR-873
bta-miR-1193
bta-miR-562
bta-miR-196b
bta-miR-150
bta-miR-329b
bta-miR-1298
bta-miR-133b
bta-miR-379
bta-miR-345-3p
bta-miR-380-3p
bta-miR-410
bta-miR-487a
bta-miR-412
bta-miR-199b
bta-miR-147
bta-miR-193a
bta-miR-122
miRNA in CC cells

C

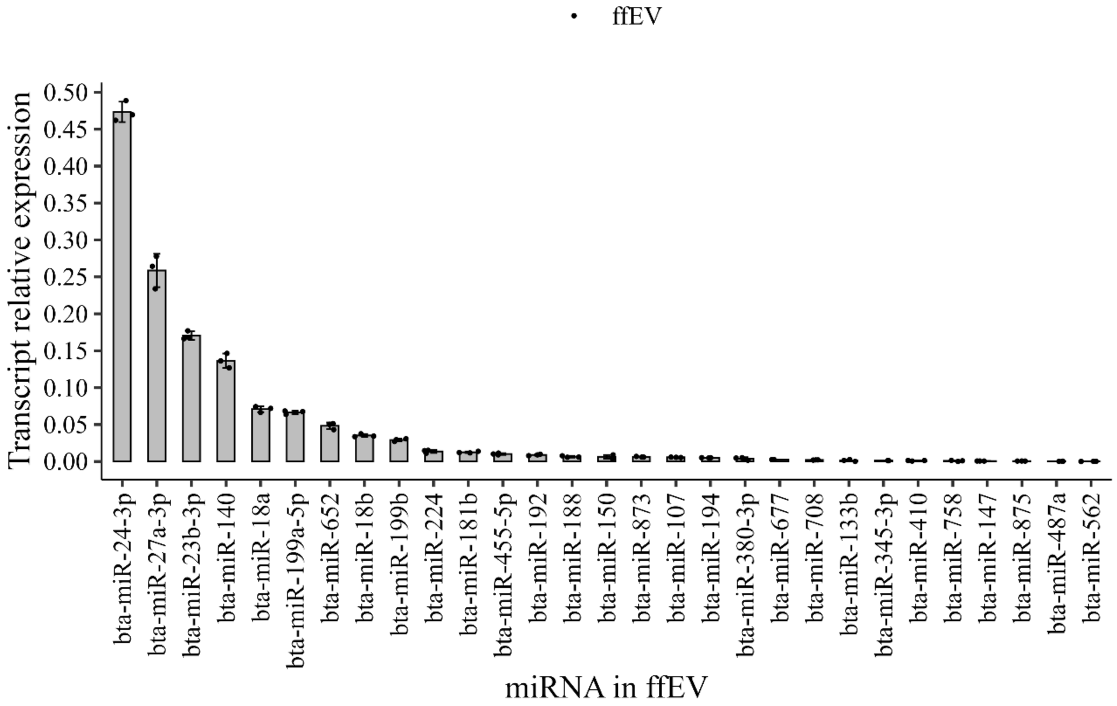
ffEV
Transcript relative expression
0.00
0.05
0.10
0.15
0.20
0.25
0.30
0.35
0.40
0.45
0.50
bta-miR-24-3p
bta-miR-27a-3p
bta-miR-23b-3p
bta-miR-140
bta-miR-18a
bta-miR-199a-5p
bta-miR-652
bta-miR-18b
bta-miR-199b
bta-miR-224
bta-miR-181b
bta-miR-455-5p
bta-miR-192
bta-miR-188
bta-miR-150
bta-miR-873
bta-miR-107
bta-miR-194
bta-miR-380-3p
bta-miR-677
bta-miR-708
bta-miR-133b
bta-miR-345-3p
bta-miR-410
bta-miR-758
bta-miR-147
bta-miR-875
bta-miR-487a
bta-miR-562
miRNA in ffEV

Supplementary Figure 4 | Characterization of ffEV. (A), (B) transmission electron microscopy demonstrating the cup shaped phenotype (100,000x magnification, 200 nm scale). (C) flow cytometry analysis of EV markers (Calcein, CD81, CD9 and Syntenin) and negative marker for cell contamination (Calnexin).

A

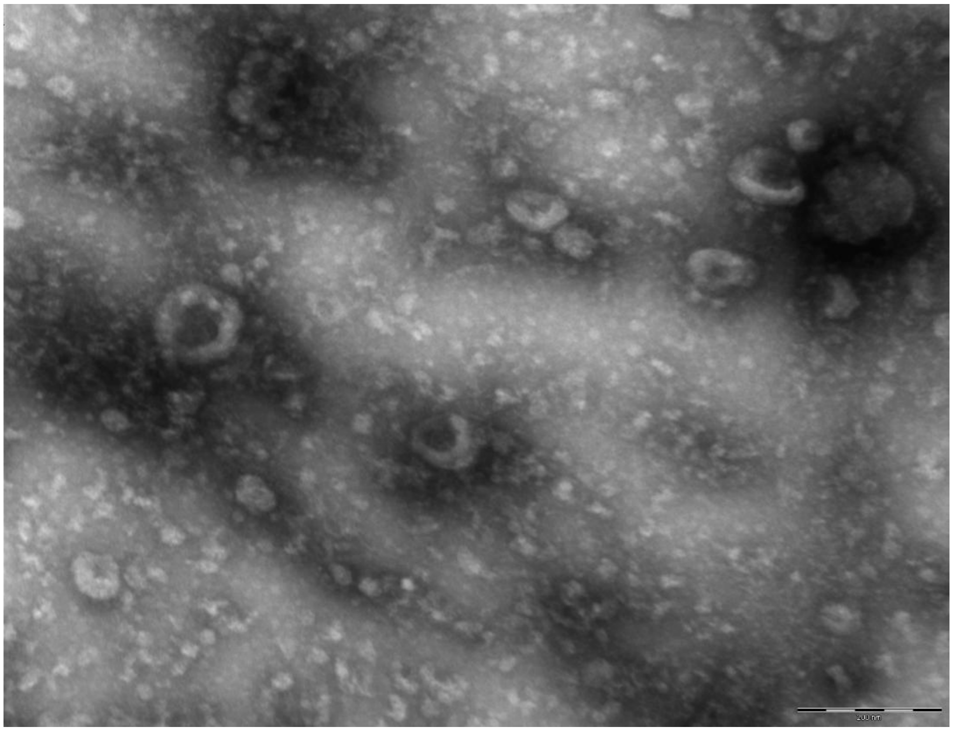

B

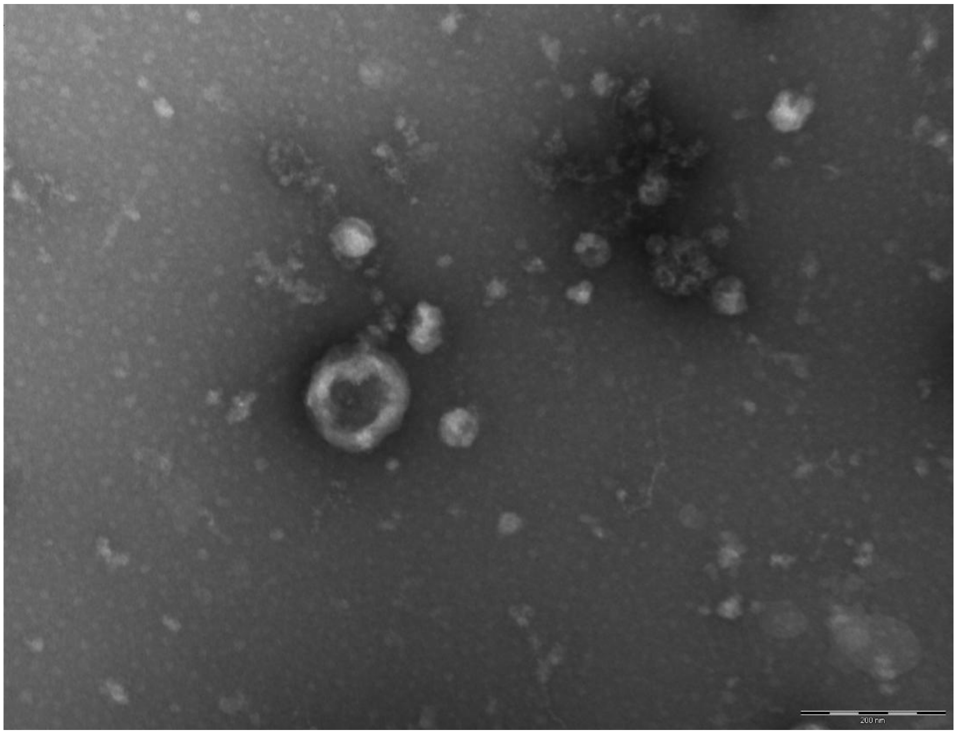

C

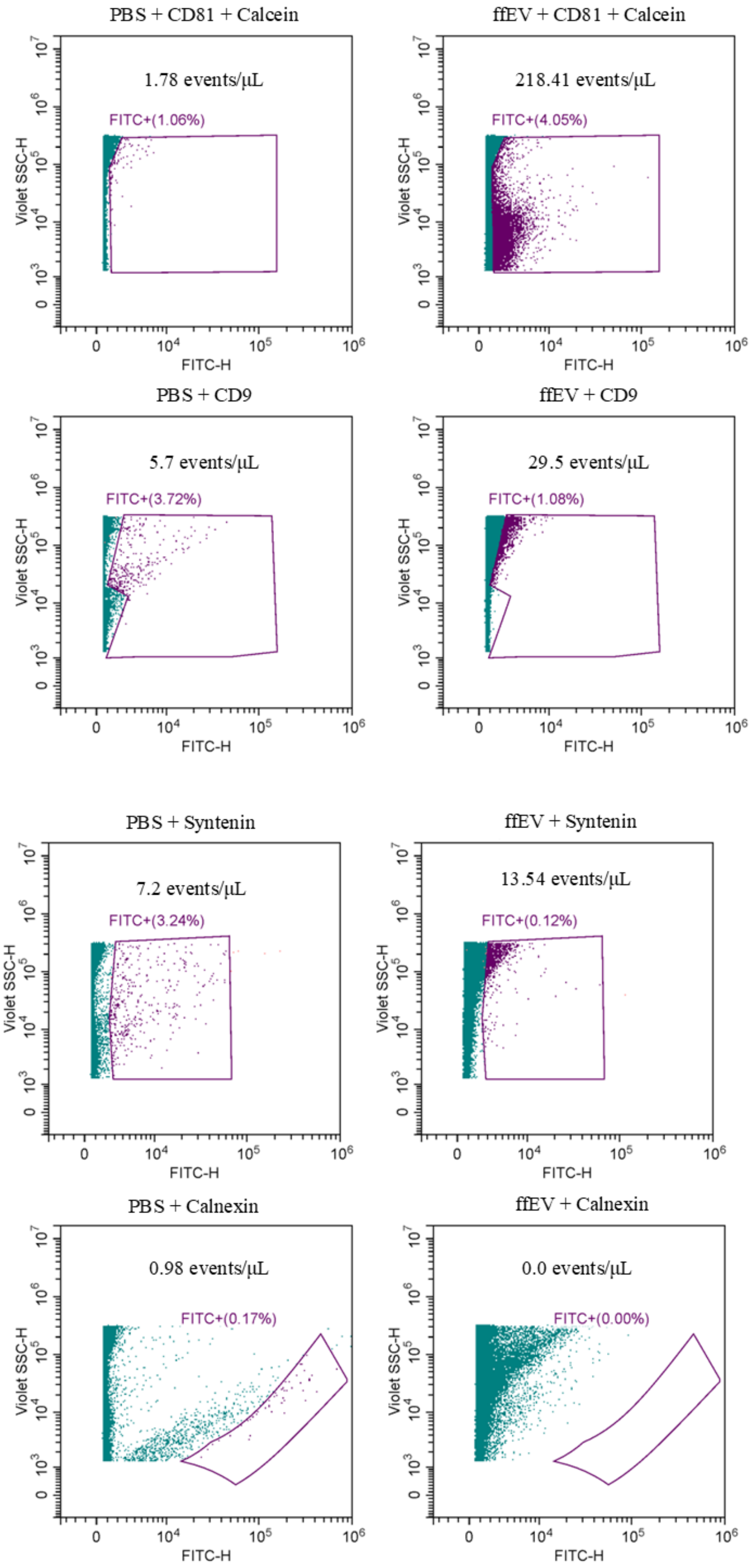
PBS + CD81 + Calcein
1.78 events/µL
FITC+(1.06%)
ffEV + CD81 + Calcein
218.41 events/µL
FITC+(4.05%)
PBS + CD9
5.7 events/µL
FITC+(3.72%)
ffEV + CD9
29.5 events/µL
FITC+(1.08%)
PBS + Syntenin
7.2 events/µL
FITC+(3.24%)
ffEV + Syntenin
13.54 events/µL
FITC+(0.12%)
PBS + Calnexin
0.98 events/µL
FITC+(0.17%)
ffEV + Calnexin
0.0 events/µL
FITC+(0.00%)
Violet SSC-H
FITC-H

Supplementary Figure 5 | Representative image of immunoblotting for detection of lipolysis protein pHSL and β-actin. MW: Molecular Weight.

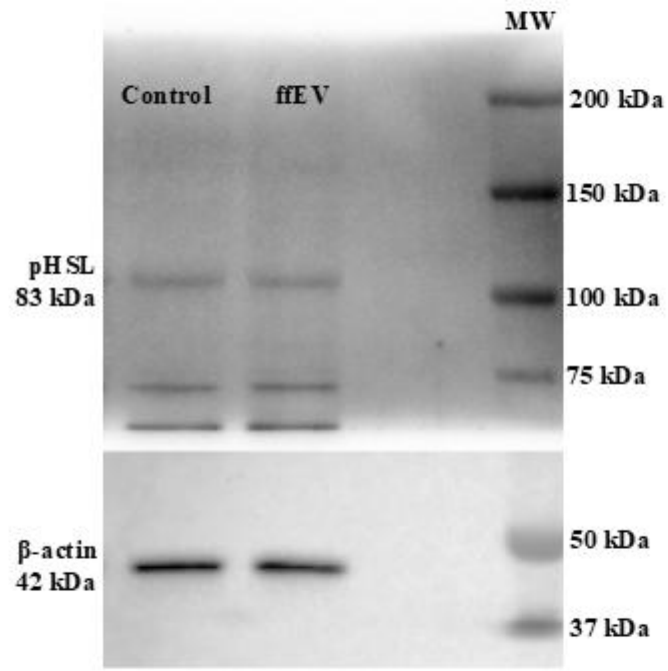

Supplement: Supplementary file 2 [file Image_1.pdf]
